# Spin-dependent thermoelectric properties of a hybrid ferromagnetic metal/quantum dot/topological insulator junction

**DOI:** 10.1038/s41598-025-87931-7

**Published:** 2025-02-10

**Authors:** Piotr Trocha

**Affiliations:** Institute of Spintronics and Quantum Information, Faculty of Physics and Astronomy, Adam Mickiewicz University, 61-614 Poznań, Poland

**Keywords:** Physics, Condensed-matter physics, Spintronics, Topological matter

## Abstract

The thermoelectric properties of hybrid system based on a single-level quantum dot coupled to a ferromagnetic metallic lead and attached to the surface states of a three-dimensional topological insulator are theoretically investigated. On the surface of a three-dimensional topological insulator, massless helical Dirac fermions emerge. We calculate the thermoelectric coefficients, including electrical conductance, Seebeck coefficient (thermopower), heat conductance, and the figure of merit, using the nonequilibrium Green’s function technique. The results are analyzed in terms of the emergence of new effects. The calculations are performed within the Hubbard I approximation concerning the dot’s Coulomb interactions. Additionally, the spin-dependent coupling of the quantum dot to the ferromagnetic lead lifts the spin degeneracy of the dot’s level, which influences the transport properties of the system. We incorporate this effect perturbatively to obtain the spin-dependent renormalization of the dot’s level. We also consider the case of finite spin accumulation in the ferromagnetic electrode, which leads to spin thermoelectric effects.

## Introduction

Spin caloritronics^1^ is a rapidly developing branch of spintronics. A breakthrough in this field was made with the discovery of the spin Seebeck effect in metallic magnets^2^. Since then, a plethora of spin thermoelectric effects have been demonstrated and investigated across various systems^3–7^, including the spin-dependent Peltier effect^8^, as well as the spin Seebeck and spin Peltier effects^9,10^ in magnetic insulator structures. Research related to the last two effects is part of the magnon spintronics field^11–14^, which primarily aims to explore methods for generating magnon currents and converting them to electronic-type spin currents, and *vice versa*. By utilizing the inverse spin Hall effect, a spin current can be transformed into a measurable electric bias voltage^15–17^. Magnon-based spintronic devices, including spin-wave multiplexers^18^ and magnon transistors^19^, have been successfully realized. Additionally, devices such as the spin Seebeck diode^20,21^, converters^22,23^, and magnon filters^24^ have been theoretically designed, presenting promising opportunities for applications in spin caloritronics.

It is well known that bulk materials exhibit a weak thermoelectric response due to constraints imposed by the Wiedemann-Franz law^25^ and the Mott relation^26^. However, reducing the dimensionality of devices can significantly enhance their thermoelectric performance^27–34^. The violation of the Wiedemann-Franz law can occur due to space quantization^35,36^, and a reduction in thermal conductance is also expected^37^. Numerous experimental^38–42^ and theoretical^43–54^ investigations on various nanoscale systems have confirmed these predictions.

As quantum dots (QDs) are considered zero-dimensional systems, extreme spatial quantization leads to a discrete energy level structure, which is expected to significantly enhance their thermoelectric response^55^. Indeed, the discrete energy level structure, along with Coulomb blockade effects^56–63^ and interference phenomena^64^, contributes to the amplification of thermoelectric efficiency.

When one of the external electrodes attached to a quantum dot (QD) is magnetic, spin-dependent thermoelectric effects can emerge^44,45,64,65^. Hybrid QD systems with a superconducting electrode^66–70^ have been investigated for their thermoelectric properties^71–76^. For instance, it has been shown that QD-based Cooper pair beam splitters can function as a heat engine or a refrigerator with efficiency close to the Carnot limit^77,78^. More recently, studies have also focused on spin-dependent thermoelectricity in these QD hybrids^79–82^. Furthermore, as it has been demonstrated that Majorana bound states (MBSs) can potentially emerge in topological superconductors (TS)^83–85^, theorists have begun exploring the thermoelectric properties of hybrid normal metal (NM)/QD/TS systems. However, most attention has been directed toward quasi-three-terminal systems, where the TS electrode is considered effectively (within the atomic limit), and thus, thermoelectricity occurs solely between the two normal metal leads^86–102^. A more realistic model of a topological superconductor^103^ allows for the description of the thermoelectric response in a two-terminal normal metal (NM)/quantum dot (QD)/TS system^104,105^.

A hybrid system can also be designed using a topological insulator. Topological insulators (TIs)^106–110^ have garnered significant interest since the first experimental verification of the topological insulator state in HgTe quantum wells^111^. The existence of two-dimensional (2D) gapless Dirac states was predicted in PbTe/SnTe and HgTe/CdTe heterostructures^112,113^ long before their experimental realization. Subsequently, theoretical models for two-dimensional TIs were proposed^114–117^. Simultaneously, it was predicted that three-dimensional (3D) TIs could be found in binary compounds involving bismuth^118–122^. The BiSb compound marked the first experimental realization of a 3D topological insulator^123,124^. These experimental and theoretical advancements triggered an avalanche of research on topological insulators, including studies on topological Dirac semimetals and topological Kondo insulators^125–130^. Later, intrinsic 3D topological insulators were demonstrated through surface transport measurements^131,132^. Furthermore, it was shown that charge currents flowing through TIs generate strong spin-transfer torque^133^, paving the way for applications in memory technology.

The BiTe compound, in addition to being a topological insulator (TI), is also a highly efficient thermoelectric material, exhibiting a high figure of merit at near room temperature^134^. This efficiency is attributed to its inherently low lattice thermal conductivity and high electronic mobility. These properties arise from the high valley degeneracy induced by strong band inversion, which connects the material’s behavior to its topological properties^135–137^.

The influence of topologically protected surface states on the thermoelectric response of various TI systems has been examined both theoretically^138–143^ and experimentally^144–146^. In contrast, the impact of bulk effects related to the topological character of the material remains relatively unexplored^147,148^. However, recent calculations have begun to illuminate the material properties that link TIs to their thermoelectric performance^149^.

On the other hand, the thermoelectric properties of quantum dot-based topological insulator systems appear to be largely unexplored^150,151^. Therefore, we investigate the thermoelectric response of a hybrid QD system with ferromagnetic (FM) and TI electrodes. This combination of external leads allows us to study spin thermoelectric effects and the influence of the TI’s surface states on the overall thermoelectric response.

The helical Dirac fermions associated with TIs exhibit intriguing properties^152–156^. In a helical metal, spins are coupled to momenta, leading to highly nontrivial magnetic properties. Furthermore, integrating both materials with a quantum dot may give rise to interesting spin-dependent effects that are reflected in the transport and thermoelectric properties of the system.

It is known that the ferromagnetism of the external electrode can induce an effective exchange field, which leads to the lifting of the spin degeneracy of the quantum dot’s energy levels^157,158^. Furthermore, this effect can influence the thermoelectric response of the system.

**Fig. 1:**
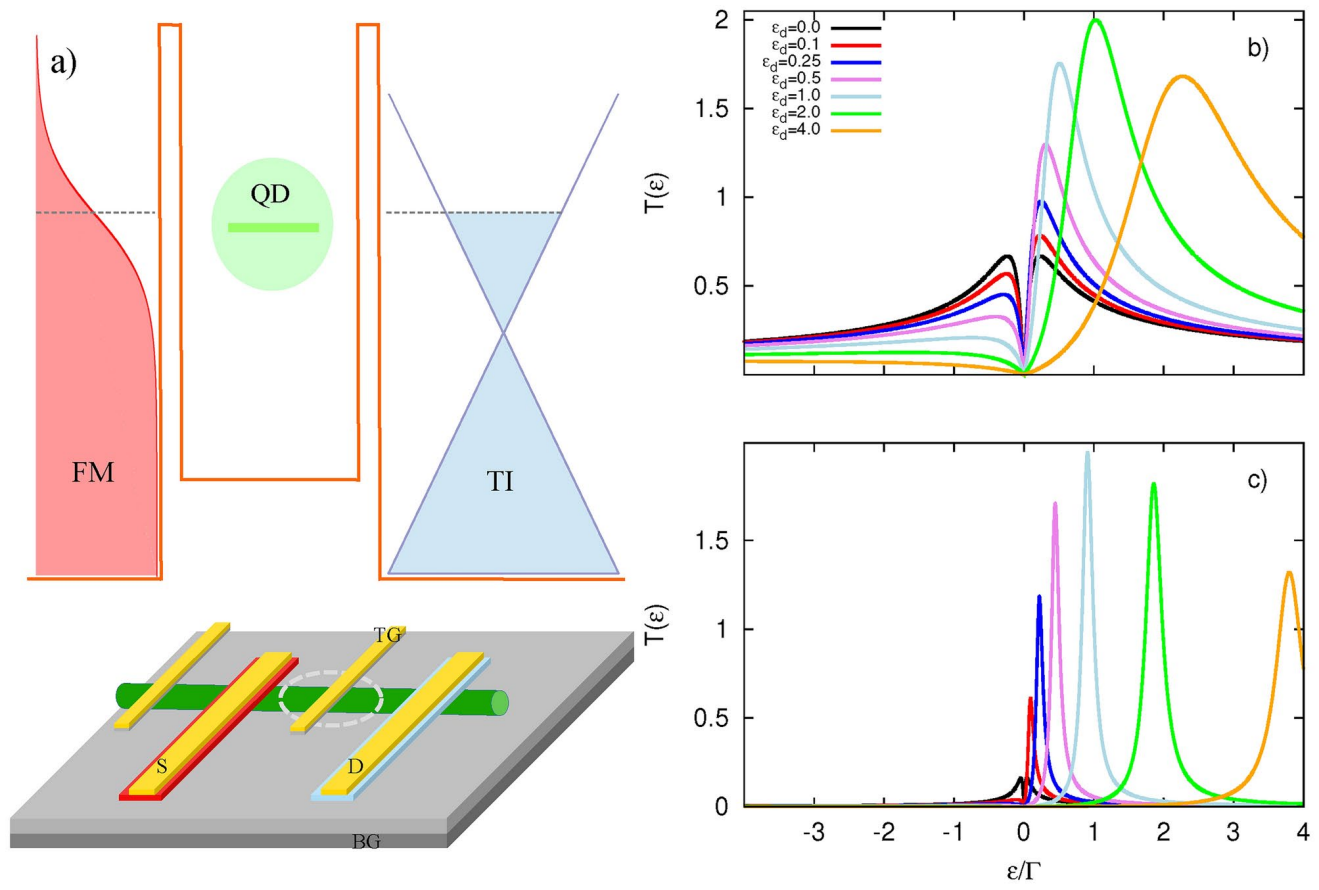
(**a**) Schematic description of the setup. (Top panel) A Fermi function (red) on the left hand side represents the ferromagnetic metal (FM), while the energy diagram of the surface states of topological insulator (TI) is depicted on the right hand side (blue). The central region (green) represents a single level quantum dot (QD), which is coupled to the two leads by tunnel couplings depicted by the two barriers surrounding the dot (orange); dashed gray lines indicate chemical potentials shown for equilibrium situation. (Bottom panel) Proposal for the experimental realization. The semiconducting nanowire or carbon nanotube (green) is placed on the semiconducting substrate surface (gray) covered by insulating layer (light gray). The substrate acts as a back-gate (BG) electrode. Source (S) and drain (D) electrodes are made of FM metal and TI material, (red and light blue) respectively, and covered by gold contacts (yellow). Top gate (TG) electrode is isolated from the nanowire by native surface oxide layer (dark gray). Applying proper BG voltage generates tunneling barriers at the contacting S and D electrodes and defines a quantum dot in the middle of the wire segment depicted by dashed-white ellipse. TG provides additional tunning of the confinement potential shape of the QD. Transmission coefficients as a function of energy calculated for the indicated values of the dot’s energy level (measured in units of $\Gamma$) and for (**b**) $\gamma _L=\gamma _R=1$, (**c**) $\gamma _L=\gamma _R=0.1$. Other parameters: $U=0$, $T=0$, $p=0$.

The paper is organized as follows. In Theoretical Background, we present the theoretical background necessary to describe the system. Specifically, we introduce the model under consideration and derive the formulae required to calculate the thermoelectric coefficients in the linear response regime. The numerical results are presented and discussed in Numerical Results, which is divided into three parts. In the first part, we examine the transmission coefficient of the system; in the second part, we describe the influence of the system’s parameters on conventional thermoelectric coefficients in the linear response regime. The final part focuses on the spin thermoelectric effects. Finally, a brief concluding section is provided in Conclusions.

## Theoretical background

### Model

We consider a system consisting of a single-level quantum dot coupled to two external leads: ferromagnetic (FM) normal metal and topological insulator emerging surface states. The setup is schematically shown in Fig. 1a, where the normal metal corresponds to the temperature smoothed Fermi function on the left, the single level QD is the central region, and the TI’s surface density of states is on the right.

The system is modeled by the Hamiltonian of the general form,

1 $$\begin{aligned} H=H_{c}+H_{QD}+H_{TL}+H_{TR}. \end{aligned}$$

The first term, $H_{c}$, describes left (L) and right (R) contacts with $H_{c}=H_L+H_R$. The Hamiltonian of the left electrode, $H_L=\sum _{{\textbf{k}}\sigma }\varepsilon _{{{\textbf{k}}}L\sigma } a^\dag _{{{\textbf{k}}}L\sigma }a_{{{\textbf{k}}}L\sigma }$, describes the metallic ferromagnet in the non-interacting quasiparticle approximation with $\varepsilon _{{{\textbf{k}}}L\sigma }$ denoting respective energy. In turn, $H_R$ stands for the Hamiltonian of the surface states of 3D TI lead described by an effective Dirac theory^159–162^,

2 $$\begin{aligned} H_R=\sum \limits _{\textbf{k}}a_{\textbf{k}}^\dag \left[ ~ \hbar v_F(\varvec{\sigma }\times \textbf{k})-\mu _0\right] a_{\textbf{k}} \end{aligned}$$

with $a_{\textbf{k}}^\dag =( a_{\textbf{k}R\uparrow }^\dag , a_{\textbf{k}R\downarrow }^\dag )$, $\textbf{k}=(k_x, k_y)$, $\varvec{\sigma }$ being a vector of Pauli matrices, $v_F$ is Fermi velocity and $\mu_0$ denoting effective chemical potential. The energy spectrum of $H_R$ is given by

3 $$\begin{aligned} E_{\pm }=\pm \hbar v_F|\textbf{k}|-\mu _0. \end{aligned}$$

After a series of transformation, which reduces the problem to an effective one-dimensional, $H_R$ can be rewritten in the form^159,160^

4 $$\begin{aligned} H_R=\sum \limits _{\sigma }\int \limits _{-k_c}^{k_c}\textrm{d}k (\tilde{\sigma }\varepsilon _{k}-\mu _0)c_{k\sigma }^\dag c_{{k}\sigma } \rightarrow \sum \limits _{\sigma }\int \limits _{-D_c}^{D_c}\textrm{d}\varepsilon \varepsilon c_{\varepsilon \sigma }^\dag c_{\varepsilon \sigma } \end{aligned}$$

with a single-particle operator $c_{k\sigma }$ ($c_{\varepsilon \sigma }$) and $\varepsilon _{k}=\hbar v_F k$ for $k=|\textbf{k}|$. Here, $\tilde{\sigma }=1$ for $\sigma =\uparrow$ and $\tilde{\sigma }=-1$ for $\sigma =\downarrow$ and $k_c$ ($D_c$) is the cutoff momentum (energy) that sets the linear dispersion region. The corresponding density of states of helical metal is

5 $$\begin{aligned} \rho _{R\sigma }(\varepsilon )=\frac{\Omega _0}{2\pi }\frac{|\varepsilon |}{(\hbar v_F)^2}\theta _{\sigma }(\varepsilon ) \end{aligned}$$

where $\theta _{\uparrow }(\varepsilon )=\theta (\varepsilon )$ and $\theta _{\downarrow }(\varepsilon )=\theta (-\varepsilon )$ with $\theta (x)$ denoting Heaviside function. It is clear that $\sigma =\uparrow$ ($\sigma =\downarrow$) correspond to the upper (lower) Dirac cone.

The second term in Eq. (1) describes quantum dot and is given by:

6 $$\begin{aligned} H_{QD}=\sum _{\sigma }\varepsilon _{d\sigma } d_{\sigma }^\dag d_{\sigma } + Un_{\uparrow }n_{\downarrow }, \end{aligned}$$

where $n_{\sigma }=d_{\sigma }^\dag d_{\sigma }$, $\varepsilon _{d\sigma }$ denotes the spin-dependent energy level of the dot, whereas *U* measures on-dot Coulomb interactions.

The next term of the Hamiltonian (1) describes the tunneling of electrons between the left lead and the dot and can be written as,

7 $$\begin{aligned} H_{T}=\sum \limits _{i=L,R}H_{Ti}=\sum \limits _{i=L,R}\sum \limits _{\textbf{k}\sigma } (V_{\textbf{k}\sigma }^i a_{\textbf{k}i\sigma }^\dag d_{\sigma }+\mathrm{H.c.}), \end{aligned}$$

with $V_{\textbf{k}\sigma }^i$ denoting the relevant tunneling matrix element. Generally, $V_{\textbf{k}\sigma }^i$ is spin- and momentum-dependent. Here, we assume that the matrix element is $\textbf{k}$ independent by taking $V_{\textbf{k}\sigma }^i\rightarrow V_{i\sigma }=\sqrt{\langle |V_{\textbf{k}\sigma }^i|^2\rangle }$ where momentum averaging is introduced by $\langle \dots \rangle$. Furthermore, we assume that the dot’s coupling to the left lead is independent of spin and can be parameterized by $\Gamma _{L}^\sigma (\varepsilon )=2\pi V_{L}^2\rho _{L\sigma }(\varepsilon )$ with $\rho _{L\sigma }(\varepsilon )$ being the relevant density of states. In the case of QD’s coupling to ferromagnetic lead, the spin dependence of $\Gamma _{L}^\sigma (\varepsilon )$ is due to spin-resolved density of states i. e. there is asymmetry between $\rho _{L\uparrow }(\varepsilon )$ and $\rho _{L\downarrow }(\varepsilon )$. In the wide-band limit $\Gamma _{L}^\sigma (\varepsilon )$ becomes energy independent, and thus, being constant, $\Gamma _{L}^\sigma (\varepsilon )=\Gamma _{L}^\sigma$ . Introducing spin polarization factor *p* for *L*th lead, we express this coupling as $\Gamma _{L}^\sigma =\Gamma _L(1+ \tilde{\sigma }p)$ with $\Gamma _L=(\Gamma _{L}^{\uparrow }+\Gamma _{L}^{\downarrow })/2$.

After a series of linear transformations tunneling Hamiltonian, $H_T$ for $i=R$, [Eq. (7)], for surface states of TI, can be written as^159,160^

8 $$\begin{aligned} H_{TR}=\sum \limits _{\sigma }\int \limits _{-D}^{D}\textrm{d}\varepsilon \left[ \sqrt{\Gamma _R(\varepsilon )}c_{\varepsilon \sigma }^\dag d_{\sigma }+\mathrm{H.c.}\right] , \end{aligned}$$

where $c_{\varepsilon \sigma }$ is the annihilation operator of an electron in the *R* lead with the (pseudo)spin index $\sigma$ at the energy $\varepsilon$ and the hybridization function $\Gamma _R(\varepsilon )=\Gamma _{R}^{\uparrow }(\varepsilon )+\Gamma _{R}^{\downarrow }(\varepsilon )$ being nonzero for all energies except at $\varepsilon =0$. The coupling of the dot to the helical metal electrode depends on energy which arises from energy-dependent density of states of the surface states [see Eq. (5)], which we clearly indicate by the $\varepsilon$-dependence. The function $\Gamma _R^{\sigma }(\varepsilon )$ is parameterized analogously like for QD’s coupling to ferromagnetic electrode resulting in $\Gamma _R^\sigma (\varepsilon )=\gamma _R^\sigma |\varepsilon |$ with $\gamma _R^\sigma =\alpha |V_{R\sigma }|^2\theta _{\sigma }(\tilde{\sigma }\varepsilon )$ and $\alpha =\Omega _0/(\hbar v_F)^2$. In the case of a spin-independent tunneling amplitude, $V_{R\sigma }\equiv V_{R}$, the total coupling simply becomes $\Gamma _R(\varepsilon )=\gamma _R |\varepsilon |$ with $\gamma _R=\alpha |V_{R}|^2$.

### Currents

Charge current through the left junction can be calculated using general formula^163^

9 $$\begin{aligned} J_e=\frac{ie}{2\hbar }\sum _{\sigma }\int \frac{{\textrm{d}}\varepsilon }{2\pi } \{[\Gamma _{L}^{\sigma }-\Gamma _{R}^{\sigma }(\varepsilon )] G_{\sigma }^{<}(\varepsilon ) +[\Gamma _{L}^{\sigma }f_L(\varepsilon )- \Gamma _{R}^{\sigma }(\varepsilon )f_R(\varepsilon )] [G_{\sigma }^{r}(\varepsilon )-G_{\sigma }^{a}(\varepsilon )]\}, \end{aligned}$$

where $G_{\sigma }^{r,a,<}$ are the retarded, advanced, and lesser Green’s functions and $f_i(\varepsilon ) = 1/\left( 1+e^{(\varepsilon -\mu _i)/T_i}\right)$ is Fermi-Dirac distribution in the *i*-th lead. Note, that due to current conservation, $J_e\equiv J_{e}^L=-J_{e}^R$.

The retarded Green’s function $\textbf{G}^{r}_{\sigma }(\varepsilon )$ can be obtained using equation of motion technique or equivalently with the help of Dyson equation

10 $$\begin{aligned} G^{r}_{\sigma }=[(g^r_{\sigma })^{-1}+\Sigma ^r_{\sigma }]^{-1}, \end{aligned}$$

with $g^r_{\sigma }$ denoting the Fourier transform of the retarded Green’s function of the QD isolated from the leads, and $\Sigma ^r_{\sigma }=\Sigma ^r_{L\sigma }+\Sigma ^r_{R\sigma }$ representing the retarded self-energy describing interaction between the QD and electrodes. The retarded Green’s function $g^{r}_{\sigma }$ of the isolated dot is derived from the relevant equation of motion. The advanced Green’s function is simply given by complex conjugation, $G^{a}_{\sigma }=[G^{r}_{\sigma }]^*$.

The retarded self-energy due to coupling to ferromagnetic lead, within the wide-band approximation, acquires the following form,

11 $$\begin{aligned} \Sigma ^{r}_{L\sigma }= -\frac{i}{2}\Gamma _{L}^{\sigma }, \end{aligned}$$

whereas the self-energy resulting from the coupling to right (TI) electrode can be obtained from the following equation:

12 $$\begin{aligned} \Sigma ^{r}_{R\sigma }=|V_{R\sigma }|^2g_{R\sigma }^{r} \end{aligned}$$

where $g_{R\sigma }^{r}$ is the retarded Green’s function for the right lead obtained by direct calculations. In the case of $V_{R\sigma }\equiv V_{R}$, $\Sigma ^{r}_{R\sigma }$ acquires the form:

13 $$\begin{aligned} \Sigma ^{r}_{R\sigma }=-\frac{\gamma _R}{2\pi }\varepsilon {\mathrm ln}\left( \frac{|D^2-\varepsilon ^2|}{\varepsilon ^2}\right) -\frac{i}{2}\gamma _R|\varepsilon |\theta (D-|\varepsilon |) \end{aligned}$$

where *D* denotes an energy cutoff for the helical metallic state. In turn, the lesser Green’s function can be obtained using the Keldysh equation,

14 $$\begin{aligned} G^{<}_{\sigma }=G^{r}_{\sigma }\Sigma ^{<}_{\sigma }G^{a}_{\sigma }, \end{aligned}$$

with the lesser self-energy

15 $$\begin{aligned} \Sigma ^{<}_{\sigma }=\Sigma ^{<}_{L\sigma }+\Sigma ^{<}_{R\sigma }= i(f_L(\varepsilon )\Gamma _{L}^\sigma + f_R(\varepsilon )\Gamma _{R}^\sigma ), \end{aligned}$$

which is valid for interactions taken in the one-particle approximations. Here, we take on-dot Coulomb interactions within Hubbard I approximation, and thus, the above formula holds.

Next, making use of identity $G^{r}_{\sigma }-G^{a}_{\sigma }=-iG^{r}_{\sigma }(\Gamma _{L}^\sigma +\Gamma _{R}^\sigma )G^{a}_{\sigma }$, the current $J_e$, Eq. (9), takes the form of a Landauer-like formula,

16 $$\begin{aligned} J_e= (J_{e\uparrow }+J_{e\downarrow })=\frac{e}{h}\sum \limits _{\sigma }\int \limits \textrm{d}\varepsilon [f_L(\varepsilon )-f_R(\varepsilon )]T_\sigma (\varepsilon ), \end{aligned}$$

where the transmission coefficient is given by $T_\sigma (\varepsilon )=\Gamma _{L}^{\sigma }\Gamma _{R}^{\sigma }|G_{\sigma }^{r}|^2$. Furthermore, spin current is defined as

17 $$\begin{aligned} J_s= \frac{\hbar }{2e}(J_{e\uparrow }-J_{e\downarrow })=\frac{1}{4\pi }\sum \limits _{\sigma }\tilde{\sigma }\int \limits \textrm{d}\varepsilon [f_L(\varepsilon )-f_R(\varepsilon )]T_\sigma (\varepsilon ). \end{aligned}$$

Following a similar derivation, one obtains the heat current flowing through left junction:

18 $$\begin{aligned} J_Q=\sum \limits _{\sigma } (J_{Q\uparrow }+J_{Q\downarrow })=\frac{1}{h}\sum \limits _{\sigma }\int \textrm{d}\varepsilon (\varepsilon -\mu _L) [f_L(\varepsilon )-f_R(\varepsilon )]T_\sigma (\varepsilon ). \end{aligned}$$

#### Linear response regime

Let us express the chemical potential and temperature of the left and right electrodes as follows: $\mu _L=\mu +\delta \mu$, $T_L=T+\delta T$ and $\mu _R=\mu$ and $T_R=T$. Then, assuming an infinitesimally small difference in bias voltage and temperature, i. e. $\delta \mu /\mu \ll 1$ and $\delta T/T\ll 1$, the spin resolved charge and heat currents become,

19a $$\begin{aligned} J_{e\sigma }=\frac{e}{h}\int \textrm{d}\varepsilon \left( -\frac{df}{d\varepsilon }\right) \left[ \delta \mu + \left( \frac{\varepsilon -\mu }{T}\right) \delta T\right] T_{\sigma }(\varepsilon ), \end{aligned}$$

19b $$\begin{aligned} J_{Q\sigma }=\frac{1}{h}\int \textrm{d}\varepsilon (\varepsilon -\mu ) \left( -\frac{df}{d\varepsilon }\right) \left[ \delta \mu + \left( \frac{\varepsilon -\mu }{T}\right) \delta T\right] T_{\sigma }(\varepsilon ). \end{aligned}$$

The linear response expressions of charge and heat currents can be cast into matrix form as:

20 $$\begin{aligned} \left( \begin{array}{c} J_{e}/e \\ J_{Q} \\ \end{array} \right) =\sum _{\sigma } \left( \begin{array}{cc} L_{0\sigma } & L_{1\sigma } \\ L_{1\sigma } & L_{2\sigma } \\ \end{array} \right) \left( \begin{array}{c} \delta \mu \\ \delta T/T \\ \end{array} \right) , \end{aligned}$$

where

21 $$\begin{aligned} L_{n\sigma }=\frac{1}{h}\int (\varepsilon -\mu )^{n}\left( -\frac{\partial f}{\partial \varepsilon }\right) T_{\sigma }(\varepsilon ). \end{aligned}$$

In turn, spin current acquires form,

22 $$\begin{aligned} J_s=\frac{\hbar }{2}\left( \sum _{\sigma }\tilde{\sigma }L_{0\sigma }\delta \mu +\sum _{\sigma }\tilde{\sigma }L_{1\sigma }\frac{\delta T}{T}\right) . \end{aligned}$$

Introducing the bias voltage drop, $\delta V=\delta \mu /e$, we are now ready to define the thermoelectric coefficients. The charge and spin conductances, *G* and $G_S$, are defined as:

23a $$\begin{aligned} & G=\left( \frac{J_e}{\delta V}\right) _{\delta T=0}=e^2\sum _{\sigma }L_{0\sigma }, \end{aligned}$$

23b $$\begin{aligned} & \quad G_s=\left( \frac{J_s}{\delta V}\right) _{\delta T=0}=\frac{e\hbar }{2}\sum _{\sigma }\tilde{\sigma }L_{0\sigma }. \end{aligned}$$

The Seebeck coefficient (thermopower), *S*, is defined as the ratio of the voltage drop $\delta V$ generated by the temperature difference $\delta T$ under the assumption of no charge current flowing through the system, $J_e=0$. Thus, one obtains:

24 $$\begin{aligned} S=-\left( \frac{\delta V}{\delta T}\right) _{J_e=0}=\frac{1}{eT}\frac{\sum _{\sigma }L_{1\sigma }}{\sum _{\sigma }L_{0\sigma }}. \end{aligned}$$

The heat conductance, $\kappa$, is the ratio of the heat current, $J_Q$, to the temperature difference, $\delta T$, in the absence of charge current:

25 $$\begin{aligned} \kappa =\left( \frac{J_Q}{\delta T}\right) _{J_e=0}=\frac{1}{T}\left( \sum _{\sigma }L_{2\sigma } -\frac{(\sum _{\sigma }L_{1\sigma })^2}{\sum _{\sigma }L_{0\sigma }}\right) . \end{aligned}$$

Finally, we introduce the figure of merit $ZT=GS^2T/\kappa$, which measures the thermoelectric efficiency of the system.

#### Spin thermoelectricity

The above formulae for thermoelectric coefficients can be used only when no spin voltage is induced in the ferromagnetic lead. However, when spin accumulation emerges due to a long spin relaxation time or by applying an external spin bias, the chemical potential of the ferromagnet becomes split. In this case, the conditions for determining the thermoelectric coefficients differs from those introduced for conventional thermoelectricity. Therefore, instead of $\delta \mu$ one must consider the spin-dependent chemical potential $\delta \mu _{\sigma }$ for each spin channel. Consequently, the Fermi-Dirac distribution function becomes spin dependent, and the currents are modified as follows:

26 $$\begin{aligned} \left( \begin{array}{c} J_e \\ J_s \\ J_Q \\ \end{array} \right) =\frac{1}{h}\sum \limits _{\sigma }\int \textrm{d}\varepsilon \left( \begin{array}{c} e \\ \tilde{\sigma }\hbar /2 \\ \varepsilon -\mu _L \\ \end{array} \right) [f_L^{\sigma }(\varepsilon )-f_R^{\sigma }(\varepsilon )]T_\sigma (\varepsilon ). \end{aligned}$$

Expressing the chemical potentials $\delta \mu _{\sigma }$ in the spin channel $\sigma$ in terms of relevant charge and spin components, $\delta \mu _{\sigma }=e\delta V_{\sigma }=\delta \mu +\tilde{\sigma }\delta \mu _{s})$, where $\delta \mu _{s} =e\delta V_s$ and $\delta V_s$ denotes spin bias voltage, the linear response expressions for charge, spin and heat currents acquire the following form:

27 $$\begin{aligned} \left( \begin{array}{c} J_{e}/e \\ 2J_{s}/\hbar \\ J_{Q} \\ \end{array} \right) =\sum _{\sigma } \left( \begin{array}{ccc} L_{0\sigma } & \tilde{\sigma }L_{0\sigma } & L_{1\sigma } \\ \tilde{\sigma }L_{0\sigma } & L_{0\sigma } & L_{1\sigma } \\ L_{1\sigma } & \tilde{\sigma }L_{1\sigma } & L_{2\sigma } \\ \end{array} \right) \left( \begin{array}{c} \delta \mu \\ \delta \mu _{s} \\ \delta T/T \\ \end{array} \right) . \end{aligned}$$

Setting $\delta \mu _s=0$, one obtains Eq. (20) and Eq. (22). The charge and spin conductances remains unchanged and are given by Eq. (23). However, the thermopower takes now the form:

28 $$\begin{aligned} S=-\left( \frac{\delta V}{\delta T}\right) _{\begin{array}{c} J_e=0 \\ J_s=0 \end{array}}=\frac{1}{2}(S_\uparrow +S_\downarrow ) \end{aligned}$$

with the thermopower in spin-$\sigma$ channel expressed as:

29 $$\begin{aligned} S_{\sigma }=\frac{1}{eT}\frac{L_{1\sigma }}{L_{0\sigma }} \end{aligned}$$

and the spin thermopower is defined as:

30 $$\begin{aligned} S_s=-\left( \frac{\delta V_s}{\delta T}\right) _{\begin{array}{c} J_e=0 \\ J_s=0 \end{array}}=\frac{1}{2}(S_\uparrow -S_\downarrow ). \end{aligned}$$

In turn, electronic contribution to the heat conductance is given now by:

31 $$\begin{aligned} \kappa =\left( \frac{J_Q}{\delta T}\right) _{\begin{array}{c} J_e=0 \\ J_s=0 \end{array}}=\frac{1}{T}\sum _{\sigma }\left( L_{2\sigma }-\frac{L_{1\sigma }^2}{L_{0\sigma }}\right) . \end{aligned}$$

where both charge and spin currents are assumed to vanish. Finally, we introduce the spin figure of merit $Z_ST=(2|e|/\hbar )(|G_s|S_sT/\kappa )$, which, along with *ZT* measures the thermoelectric efficiency of the system.

## Numerical results

In this section we present numerical results obtained from the formulae introduced above. This section is divided into two parts. In the first part, results on conventional thermoelectricity are presented, whereas in the second part, we show numerical results obtained when spin accumulation in the ferromagnetic electrode is relevant. In numerical calculations, we assume spin independent TI’s tunneling amplitude ($V_{R}$), and thus, $\Gamma _R=\gamma _R |\varepsilon |$, where $\gamma _R$ is dimensionless coefficient introduced in Model. Moreover, we introduce similar parameter for dot’s coupling to FM (left) electrode in the following way, $\Gamma _L=\gamma _L\Gamma$. Such parametrization allows to tune both couplings independently. Typical values of the dot’s tunnel coupling to external electrodes range from $\mu$eVs to approximately 10 meV^164,165^. Consequently, the parameter $\gamma _i$ can vary from 0.01 to as high as 100 by adjusting the size of the tunneling barrier. The parameter $\Gamma$ can be set as $\Gamma = 0.1$ meV, which provides an experimentally accessible coupling range for $\Gamma _L$, spanning from 1 $\mu$eV for $\gamma _L = 0.01$ to 10 meV for $\gamma _L = 100$. Current experimental techniques have demonstrated electrically tunable tunnel couplings ranging from less than 1 $\mu$eV to over 600 $\mu$eV^166^. This suggests that $\gamma _i$ can be tuned from 0.01 to 6 purely through electrostatic means, without altering the architecture of the tunnel barrier. In the following all energy quantities will be expressed in units of parameter $\Gamma$. Furthermore, we assume spin-degenerate dot’s energy level $\varepsilon _{d\uparrow }=\varepsilon _{d\downarrow }\equiv \varepsilon _d$, if not stated otherwise. We will separately consider the cases of zero on-dot Coulomb energy and a finite value of the parameter *U*.

**Fig. 2:**
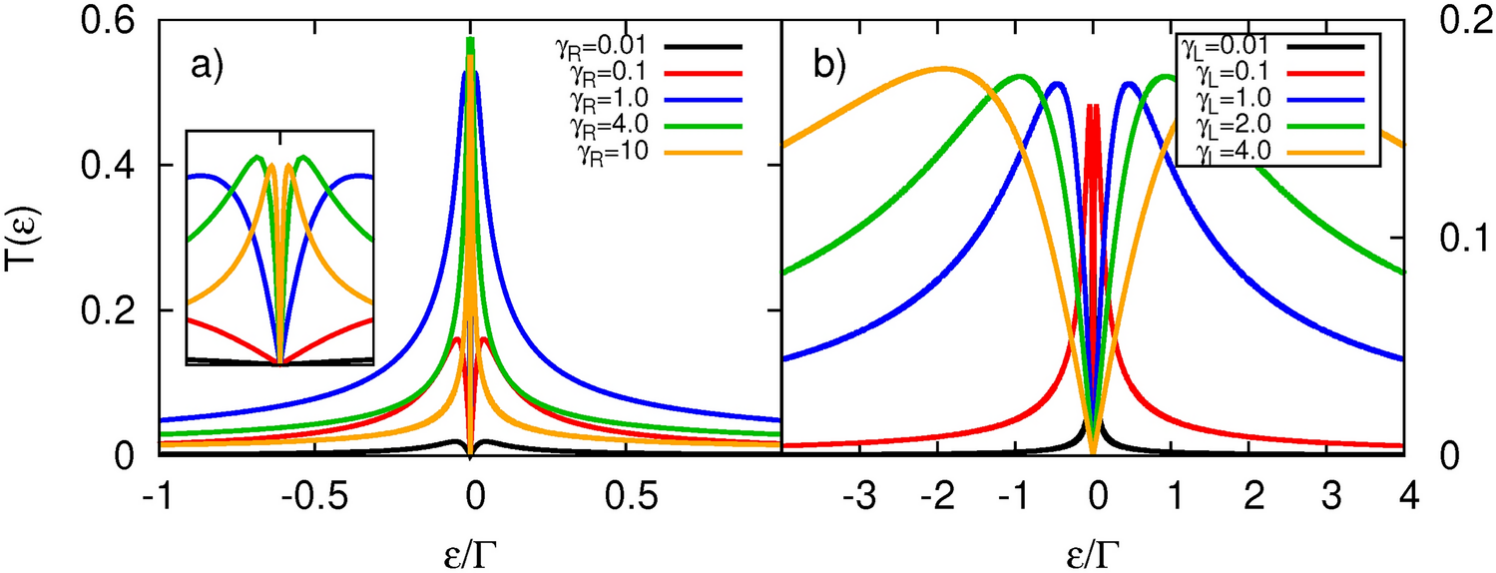
Transmission coefficients as a function of energy calculated for indicated values of the coupling parameters and for $\varepsilon _d=0$. Left panel (**a**) shows results for $\gamma _L=0.1$ while varying $\gamma _R$, whereas right panel (**b**) presents situation for $\gamma _R=0.1$ while varying $\gamma _L$. Insets show a zoomed-in view of the central peaks region with the displayed energy range being $\varepsilon /\Gamma \in [-0.02,0.02]$. Other parameters: $T=0$, $U=0$, $p=0$.

### Transmission coefficient

In this section, we investigate the influence of system parameter variations on the transmission coefficient introduced in Currents. In the following, we assume chemical potential to be grounded, i. e. we set $\mu =0$. In Fig. 1b,c), the transmission is shown for different values of the dot’s energy level. First, the transmission function exhibits a dip at $\varepsilon =0$ regardless of the dot’s energy level, and the transmission vanishes at the center of the dip, i. e. , at $\varepsilon =0$. This dip is a direct consequence of the vanishing density of states of the TI’s surface states at zero energy, which prevents the transfer of electrons at the Fermi level from the FM electrode. As the dot’s energy level increases, the transmission becomes asymmetric, unlike the case for $\varepsilon _d=0$. The intensity of $T(\varepsilon )$ for positive energy $\varepsilon >0$ grows with increasing $\varepsilon _d$, reaching unity for each spin channel at sufficiently high dot energy levels. This behavior is well understood when recalling that the TI’s density of states increases linearly with energy. Thus, as the dot’s energy level moves further above the Fermi level, more electrons can propagate through the QD to the empty states of the TI, whose number increases with energy. However, this holds only up to a certain dot’s energy level, beyond which the transmission starts to gradually decrease as $\varepsilon _d$ increases further. This decrease is due to the reduced population of high-energy electrons in the normal electrode, as governed by the Fermi-Dirac distribution (see Fig. 1a). In contrast, the amplitude of the transmission for $\varepsilon <0$ correspondingly decreases.

Moreover, the position of the maximum doesn’t follow the dot’s energy level directly for relatively strong coupling to the TI, as shown in Fig.1b. This feature can be attributed to the strong renormalization of the dot’s energy level due to the real part of the self-energy [given by Eq.(12)], which becomes more relevant for stronger coupling of the QD to the TI. In turn, for weaker coupling strength (Fig.1c), the maximum of the transmission follows the dot’s energy level position. Additionally, the width of the transmission peak also grows with $\varepsilon _d$, as a consequence of the specific energy dependence of the TI’s imaginary part of the self-energy [Eq.(12)]. Note that so far we have assumed a non-magnetic left electrode, and therefore, the transmission coefficients for both spin components are the same.

In Fig. 2a, the transmission coefficient is presented for a fixed value of $\gamma _L$, while varying the value of the parameter $\gamma _R$. When the QD is weakly coupled to the TI electrode, $T(\varepsilon )$ becomes strongly suppressed. An increase in the dot’s coupling strength to the TI leads to a rise in transmission intensity, as expected. However, for extremely strong coupling, represented by parameter $\gamma _R$, the amplitude begins to drop. In turn, the width of the transmission profile decreases with increasing $\gamma _R$, indicating stronger localization of the dot’s level. Regarding Fig. 2b, one can observe that the dot’s coupling strength to the normal metal electrode significantly affects the width of the transmission function, while only slightly influencing its intensity.

**Fig. 3:**
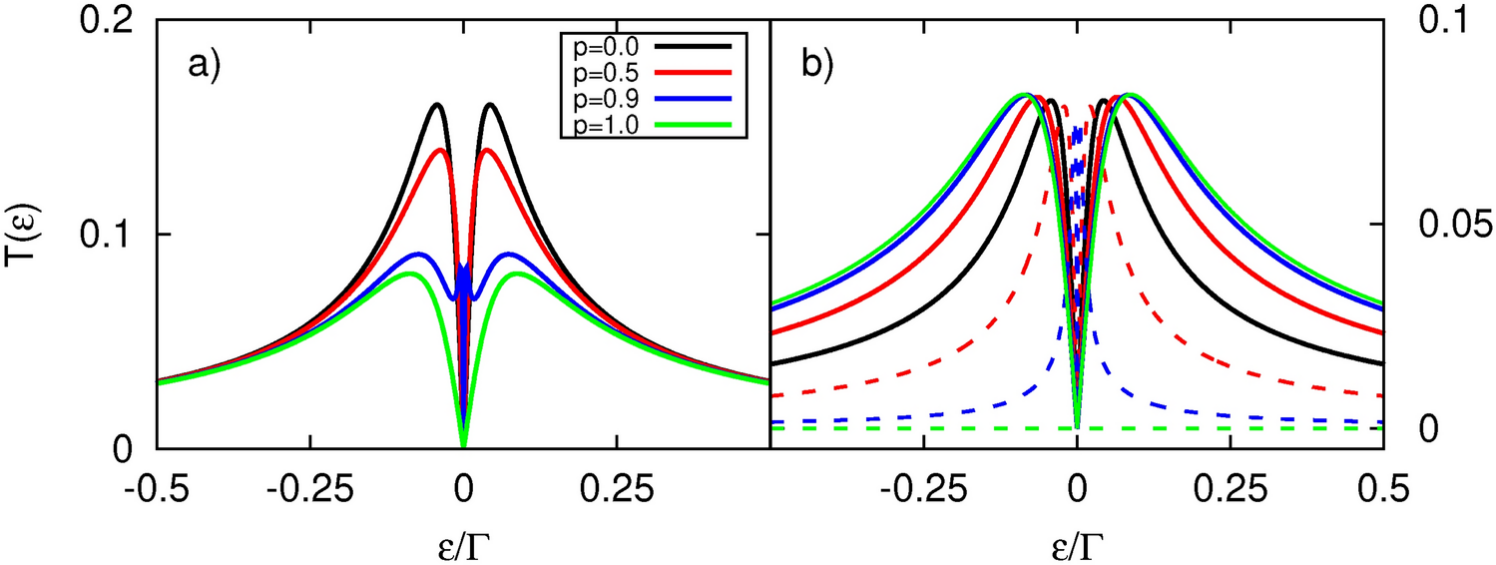
Transmission coefficients as a function of energy calculated for indicated values of spin polarization factor *p* and for $\varepsilon _d=0$. Left panel (**a**) shows total transmission, whereas right panel (**b**) displays spin up (solid line) and spin down (dashed line) transmission components. Note that the dashed black curve is overlaid by the solid black curve for $p=0$. Other parameters: $\gamma _L=\gamma _R=0.1$, $U=0$, $T=0$.

The magnetic properties of the FM (left) electrode can also significantly influence the transmission. Specifically, an increase in the spin polarization factor *p* leads to a gradual decrease in the transmission coefficient $T(\varepsilon )$, with its maximum value halved for a fully spin-polarized left electrode ($p=1$) (see Fig. 3a). In this case, only electrons with spin-up orientation participate in transport. Although the density of states for spin-up electrons increases with *p*, the number of states in the TI electrode remains the same for both spin components. At the same time, the spin-down component of the dot’s coupling to the ferromagnet, $\Gamma _L^\downarrow$, and the corresponding component of the density of states, decreases with increasing *p*. This results in the aforementioned behavior of $T(\varepsilon )$. To provide better visualization, we also present the spin-resolved transmission coefficients in Fig. 3b. It is clear that for finite *p*, spin-up electrons are the majority carriers driving the transport, while spin-down electrons become relevant at low temperatures. Notably, a two-peak structure of $T_{\downarrow }(\varepsilon )$ emerges within the dip of $T_{\uparrow }(\varepsilon )$. Moreover, sufficiently far from $\varepsilon =0$, the transmission reaches the same values regardless of *p*, as the overall coupling to the FM remains constant irrespective of *p*. In turn, the width of the transmission profile increases with rising *p*. This behavior differs from that observed in the FM/QD/NM setup, where the total width of $T(\varepsilon )$ is largely unaffected by changes in *p*, or even slightly decreases.

### Conventional thermoelectricity

Here, we present results associated to the thermoelectric response of the system in the linear response regime, in the absence of spin bias. Particularly, we study quantities, including the electrical conductance, Seebeck coefficient known also as thermopower, the heat conductance and the corresponding figure of merit, introduced in Linear response regime.

**Fig. 4:**
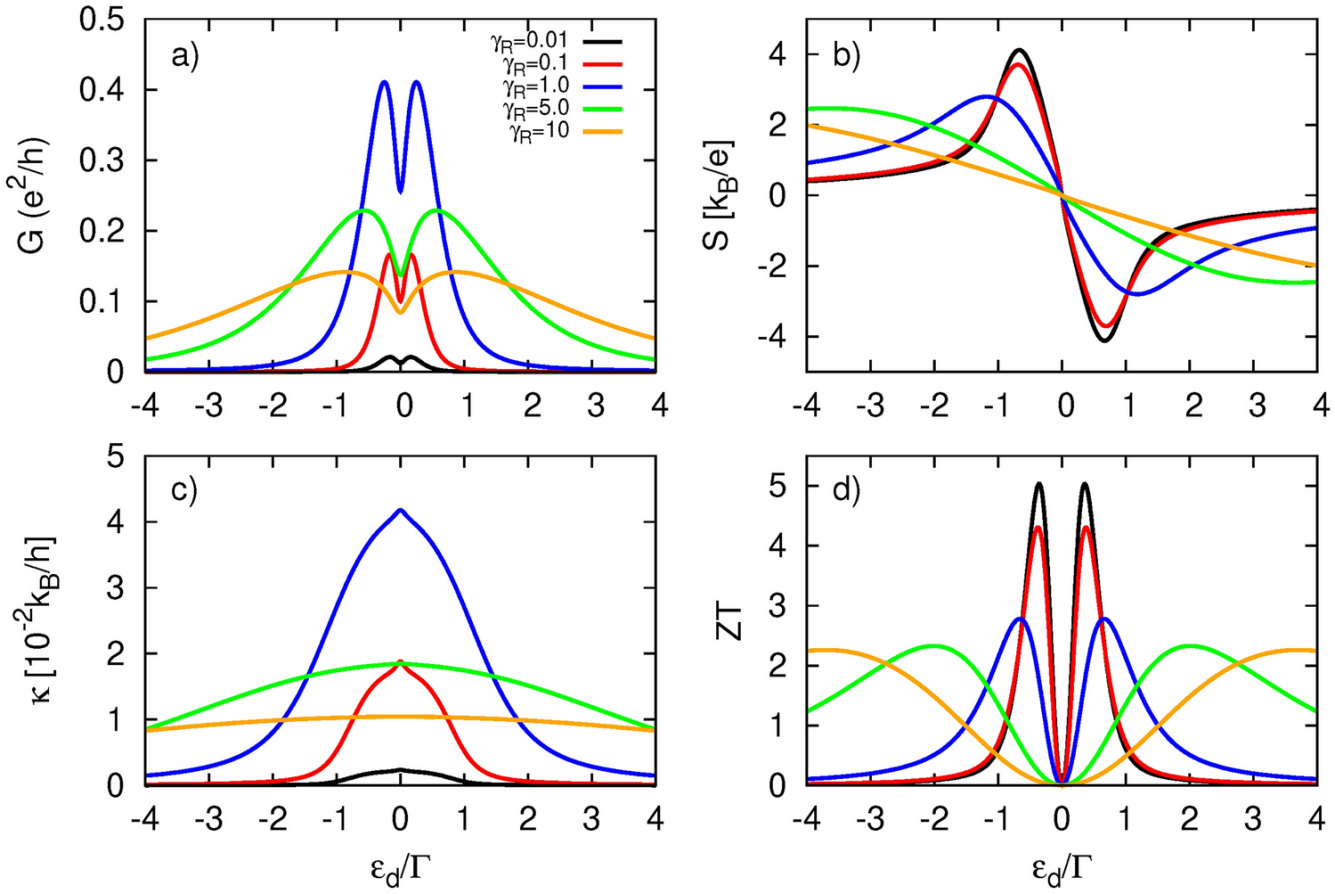
Thermoelectric coefficients: (**a**) electrical conductance, (**b**) Seebeck coefficient (thermopower), (**c**) heat conductance, (**d**) figure of merit, calculated as a function of the dot’s energy level $\varepsilon _d$ and for indicated values of parameter $\gamma _R$. The other parameters are: $\gamma _L=0.1$, $k_B T=0.1\Gamma$, $U=0$, $p=0$. Here and in the following figures $e=|e|$.

To better understand the results for the general case, let us first consider the limit $U=0$. It is also instructive to consider first the case of nonmagnetic lead. Therefore, we first discuss the case where $p=0$. In Fig.4, we present the thermoelectric coefficients as a function of the dot’s energy level, $\varepsilon _d$, for the indicated values of the asymmetry coupling parameter $\gamma _R$. The electrical conductance, shown in Fig.4a, reveals nonmonotonic behavior as the dot’s coupling strength to the TI electrode increases. First, the electrical conductance mirrors the behavior of the transmission function, e. g. , the dip structure observed in the transmission also appears in the electrical conductance. Second, for small $\gamma _R$, the electrical conductance is significantly suppressed, a result of the corresponding $T(\varepsilon )$ dependence (see Fig. 2a). Increasing $\gamma _R$ leads to a rise in the*G*’s amplitude, reaching a maximum value at a specific $\gamma _R$, namely $\gamma _R^{\text {max}} \approx 0.9$. Further increases in $\gamma _R$ beyond $\gamma _R^{\text {max}}$ result in a reduction of the electrical conductance maxima. This behavior of *G* can be understood by considering the transmission coefficient (Fig.4a) and the profile of the derivative ($\textrm{d}f/\textrm{d}\varepsilon$) of the Fermi function. The width of the latter is relatively broad at the assumed temperature, placing the main features of $T(\varepsilon )$ within the range of the $\textrm{d}f/\textrm{d}\varepsilon$ width. Note that electrons within this range contribute to transport. When $\gamma _R$ is small, the amplitude of $T(\varepsilon )$ is strongly suppressed, and its dip is broad, which explains the low electrical conductance. However, as $\gamma _R$ increases, the amplitude of $T(\varepsilon )$ grows and the dip narrows, leading to an increase in *G*. Simultaneously, the increase in $\gamma _R$ results in a narrowing of the transmission resonance width, while its intensity depends weakly on $\gamma _R$ for $\gamma _R>1$. Thus, beyond $\gamma _R^{\text {max}}$, fewer electrons are transferred, and the electrical conductance begins to decrease. In turn, the width of *G* becomes broader as $\gamma _R$ increases, which is related to the dependence of $T(\varepsilon )$ on $\gamma _R$ and $\varepsilon _R$. Specifically, for $\varepsilon _d \ne 0$, the transmission peak is situated at an energy equal to $\varepsilon =\varepsilon _d$ only for sufficiently small $\gamma _R$, as explained in the previous section. For $|\varepsilon _d| \gg 0$, only the tail of the transmission peak lies within the $\textrm{d}f/\textrm{d}\varepsilon$ peak, contributing to transport. An increase in $\gamma _R$ leads to a narrowing of $T(\varepsilon )$, as explained above, but it also results in stronger renormalization of the dot’s energy level, pushing it toward zero energy. Thus, for sufficiently strong coupling to the TI electrode, the entire transmission peak falls within the range of the $\textrm{d}f/\textrm{d}\varepsilon$ resonance, explaining the slow decrease of *G* as $|\varepsilon _d|$ increases. In Fig. 4c, we show the heat conductance $\kappa$, which exhibits a broad maximum centered at $\varepsilon _d=0$. Its amplitude behaves similarly to that of the electrical conductance.

The Seebeck coefficient, shown in Fig. 4b, changes sign at $\varepsilon _d=0$ because no net charge current is generated by the temperature difference. Consequently, no bias voltage is built, and the thermopower vanishes. More precisely, at resonance, the charge current due to electrons is completely compensated by that associated with holes. It is also noticeable that for $\varepsilon _d < 0$, the thermopower is positive, as the majority carriers are holes. Conversely, when the dot’s energy level is above the Fermi level, electrons become the main carriers, resulting in negative thermopower.

Generally, the maxima of |*S*| decrease with increasing dot coupling strength to the TI. However, for larger $\gamma _R$, the thermopower (|*S*|) shows relatively high values over a broader range of $\varepsilon _d$. The behavior of *S* can be explained by considering the corresponding electrical conductance and recalling that the Seebeck coefficient is defined under the condition of vanishing charge current. Therefore, when *G* is small, a relatively large bias voltage is required to compensate for the thermally induced charge current. As a result, the thermopower exhibits the largest maximum values when $\gamma _R \ll 1$. As the dot’s coupling to the TI increases, it becomes easier to transfer heat between the leads, i. e. the $L_1$ coefficient increases, which might suggest a rise in |*S*|. However, at the same time, the electrical conductance increases (at faster rate), leading to a reduction in thermopower. It is important to note that the maximum of |*S|* shifts away from $\varepsilon _d=0$ as $\gamma _R$ increases. This shift is due to the competition between the *G* ($L_0$) and $L_1$ coefficients. The larger the $\gamma _R$, the broader the conductance structure, meaning the dot’s level must be tuned further from zero energy to maximize *S* when $L_1$ outweighs $L_0$. As a result, *S* can achieve relatively large values far from resonance when $\gamma _R \gg 1$, as the conductance is suppressed sufficiently. However, further increases in $\varepsilon _d$ lead to a suppression of |*S*|. This occurs regardless of the value of $\gamma _R$ and is due to the reduction in charge (and, consequently, heat) transfer when $\varepsilon _d$ is far from zero energy.

The behavior of electrical conductance, heat conductance, and the Seebeck coefficient leads to the figure of merit shown in Fig. 4d. It is noteworthy that the largest overall thermoelectric response occurs when the QD is relatively weakly coupled to the TI electrode, with $ZT > 4.5$ for $\gamma _R \le 0.1 = \gamma _L$. Conversely, significant values of *ZT* (approximately $ZT \gtrsim 2$) are also attained for $|\varepsilon _d| \gg 0$ when $\gamma _R \gg 1$.

**Fig. 5:**
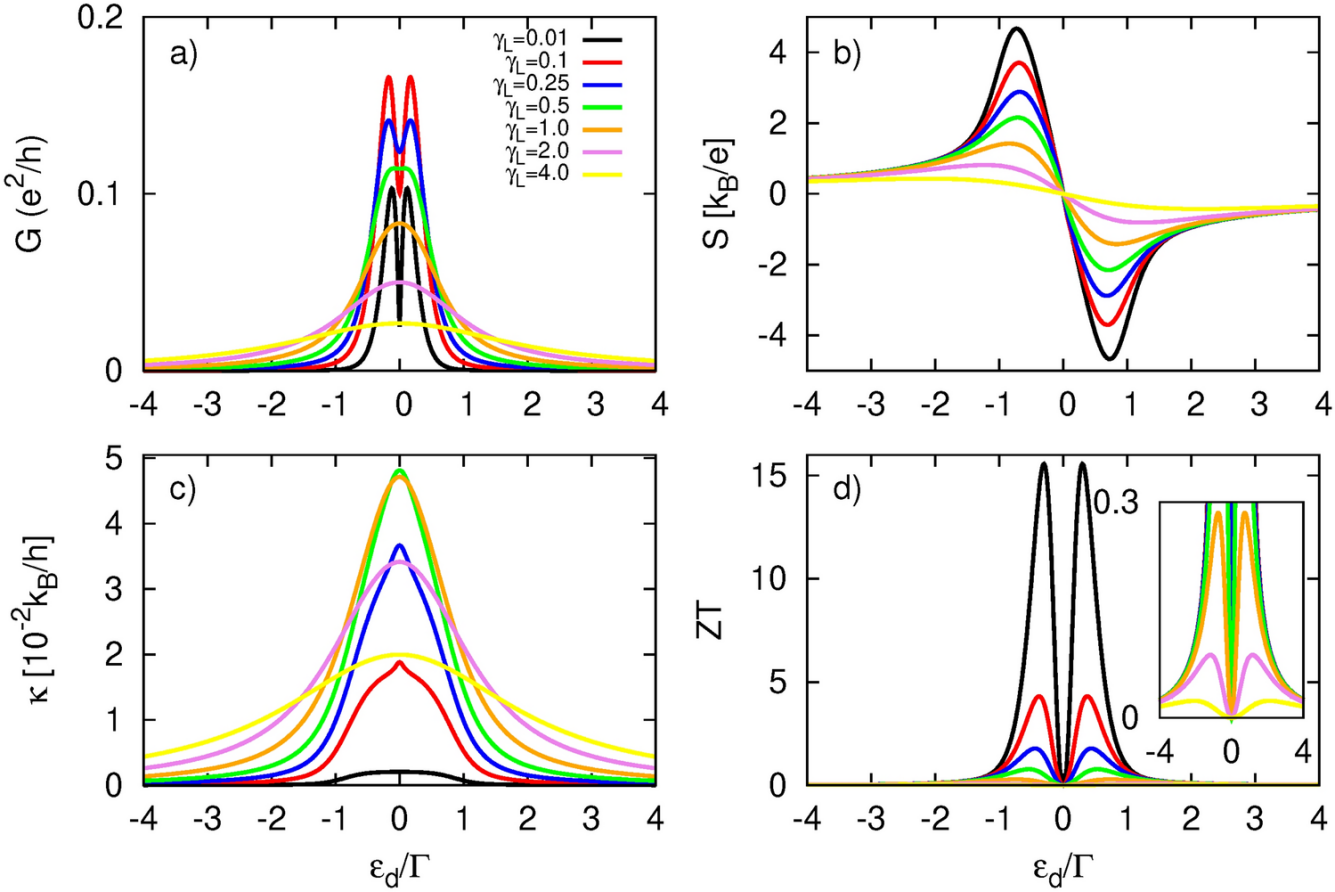
Thermoelectric coefficients: (**a**) electrical conductance, (**b**) Seebeck coefficient (thermopower), (**c**) heat conductance, (**d**) figure of merit, calculated as a function of the dot’s energy level $\varepsilon _d$ and for indicated values of parameter $\gamma _L$. The other parameters are: $\gamma _R=0.1$, $k_B T=0.1\Gamma$, $p=0$, $U=0$. Inset in d) shows a zoomed-in view of the central peaks region.

Now, we analyse the influence of dot’s coupling to the normal metal on the system’s thermoelectric response. The amplitude of electrical conductance *G*, presented in Fig. 5, reveals a nonmonotonic dependence on $\gamma _L$. One notices that the characteristic two-peak structure in the electrical conductance persists only for relatively weak coupling to NM electrode, i. e. for $\gamma _L<0.5$. In turn, for $\gamma _L\approx 0.5$, the two peaks merge into one, and its width grows with further increase in the dot’s coupling to the normal metal electrode. These features can be attributed to the broadening effect of the dot’s level, as the width of the QD’s level is directly related to the coupling strength. Let us now consider *G* for $\varepsilon _d$ close to zero to explain its behavior in more detail. Weak coupling to NM electrode doesn’t lead to such strong suppression of *G* as noticed for the corresponding coupling to the TI reservoir. Electrical conductance *G* grows with increasing $\gamma _L$, achieving maximum for $\gamma _L\approx \gamma _R$ which can be explained referring to the transmission coefficient shown in Fig. 2b. The width of transmission peak structure gradually increases with $\gamma _L$, but its amplitude remains roughly constant. As long as this broadening occurs within the energy range defined by the width of the derivative of the Fermi function, $\textrm{d}f/\textrm{d}\varepsilon$, the electrical conductance rises. When $\gamma _L$ reaches the critical value $\gamma _L^{max}$, the electrical conductance achieves its maximum and then diminishes with further increases in $\gamma _L$. The drop of *G* is due to a lesser portion of the transmission being located within the range defined by the width of $\textrm{d}f/\textrm{d}\varepsilon$ - the tails of $T(\varepsilon )$ simply moves outside of this range. Moreover, the dip structure of $T(\varepsilon )$ widens, which further reduces the transmission within the $\textrm{d}f/\textrm{d}\varepsilon$ width. Both effects lead to a reduction in the number of transferred electrons, and thus to a decrease in *G*. In turn, heat conductance $\kappa$ (Fig. 5c) reveals a similar behavior to *G*, but the maximum amplitude is achieved at greater value of $\gamma _L$. Interestingly, $\kappa$ is maximized for the same value of $\gamma _L$ at which electrical conductance loses its two-peak structure due to level broadening.

In Fig. 5b, we show Seebeck coefficient, which increases with $\gamma _L$ for all dot energy levels except at $\varepsilon _d=0$, where it vanishes. This behavior can be associated to the competition between $L_0$ and $L_1$ components. Both quantities reveal a nonmonotonic dependence on $\gamma _L$: starting from $\gamma _L=0$, both $L_0$ and $|L_1|$ increase, reaching a maximum before decreasing with further increases in $\gamma _L$. However, $L_0$ and $|L_1|$ change at different rates: $L_0$ increases faster than $|L_1|$ before reaching its maximum and subsequently decreases more slowly than $|L_1|$. The detailed $\gamma _L$-dependence of $L_0$ and $|L_1|$, shown in the SI,  confirms this behavior. As a consequence, the absolute value of thermopower decreases with $\gamma _L$ as a smaller bias voltage is required to compensate the charge current induced by the temperature difference between the electrodes. The resulting *ZT*, shown in Fig. 5d, strongly depends on variation in $\gamma _L$, increasing as the coupling strength to the NM reservoir decreases, achieving remarkable values for sufficiently small $\gamma _L$. Specifically, $ZT>1$ for $\gamma _L\le 0.41$, and *ZT* can reach exceptionally high values, exceeding 15, for $\gamma _L\ll \gamma _R$, e. g. $ZT\approx 15.6$ for $\gamma _L=0.01$.

**Fig. 6:**
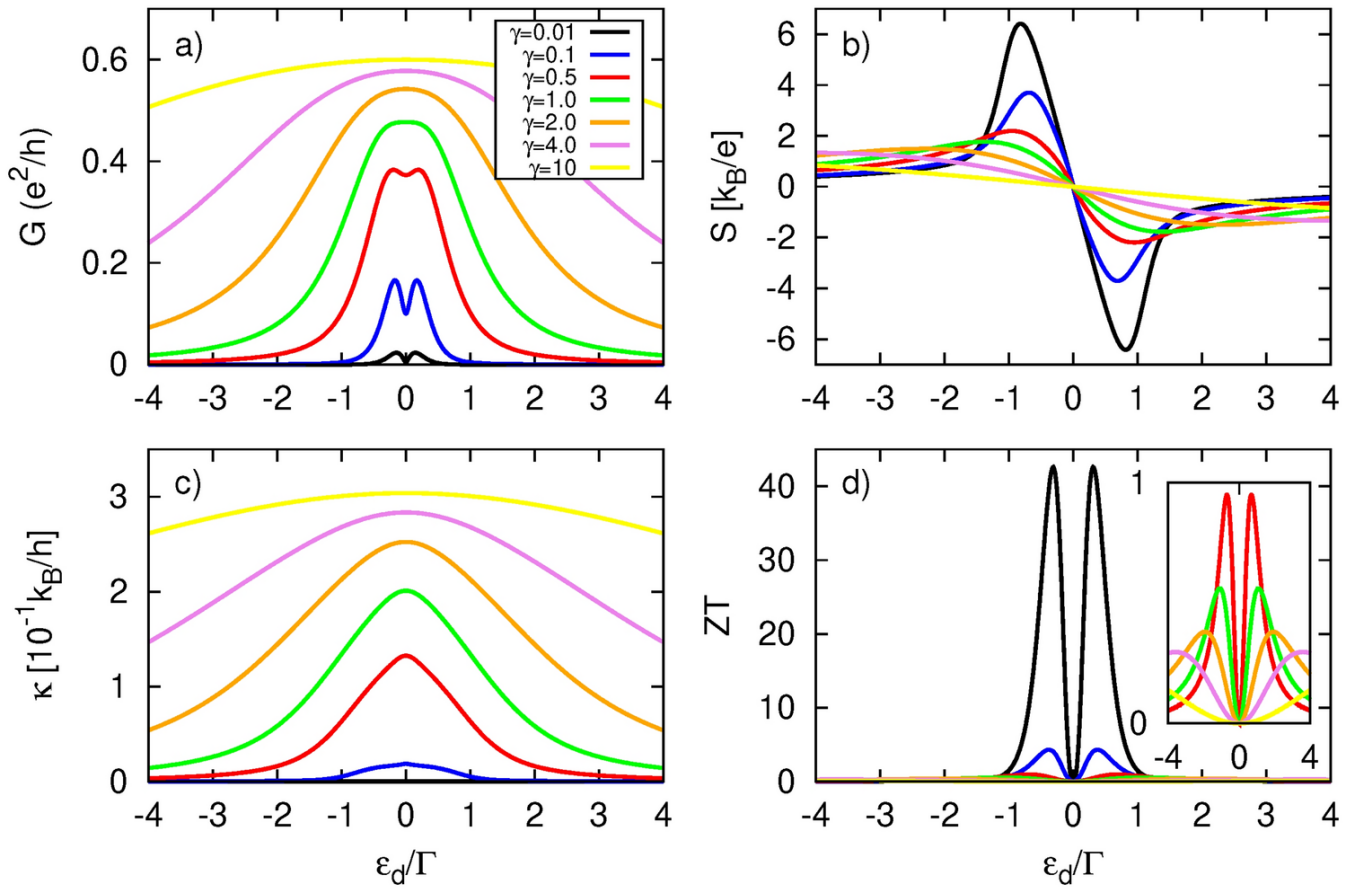
Thermoelectric coefficients: (**a**) electrical conductance, (**b**) Seebeck coefficient (thermopower), (**c**) heat conductance, (**d**) figure of merit, calculated as a function of the dot’s energy level $\varepsilon _d$ and for indicated values of parameter $\gamma _L=\gamma _R=\gamma$. Inset in d) shows a zoomed-in view of the central peaks region. The other parameters are: $k_B T=0.1\Gamma$, $p=0$, $U=0$.

To compare the results for the considered system with those for a quantum dot coupled to two normal metal electrodes, the relevant calculations have been performed and are presented in Fig. 7 in the SI. It is evident that the presence of the surface states of the TI electrode enhances the thermoelectric response, as both |*S*| and *ZT* achieve larger values. Notably, *ZT* can be orders of magnitude larger for the present system than for a quantum dot coupled to two normal leads under the same parameter set. Additionally, the *ZT* presented in Fig. 4d) exhibits a much weaker dependence on $\gamma _R$ compared to that shown in Fig. 7d in the SI for the normal metallic system. Furthermore, in the normal metallic system, *ZT* also decreases much more rapidly with $\gamma _L$.

For completeness, Fig. 6 presents the thermoelectric coefficients for different values of the parameter $\gamma \equiv \gamma _L = \gamma _R$. Here, the couplings of the QD to both electrodes are tuned simultaneously. However, it is important to note that even for $\gamma _L = \gamma _R$, the system is not symmetric due to the energy dependence of the QD’s coupling to the surface states of the TI. In other words, the proportionality $\Gamma _L = \lambda \Gamma _R$, where $\lambda$ is a constant, does not hold. Both electrical and heat conductance, shown in Fig. 6a,c, respectively, increase with $\gamma$, as the system becomes progressively transparent over a broader energy range of tunneling electrons. In turn, the Seebeck coefficient (Fig. 6b) exhibits behavior quite similar to that observed in Fig.4b, highlighting the significant role of the dot’s coupling to the TI electrode in generating a voltage drop due to the temperature difference between the leads. However, the simultaneous adjustment of both couplings leads to an even more pronounced *ZT* (Fig.6d) compared to when the couplings are tuned independently-see Figs. 4d, 5d for comparison. In the weak coupling regime, *ZT* achieves values exceeding 40. Although *ZT* is also enhanced for small dot couplings in the case of a normal metal system (see Fig. 8d in the SI), the resulting *ZT* remains several times smaller than that obtained for the considered system under the same parameters.

**Fig. 7:**
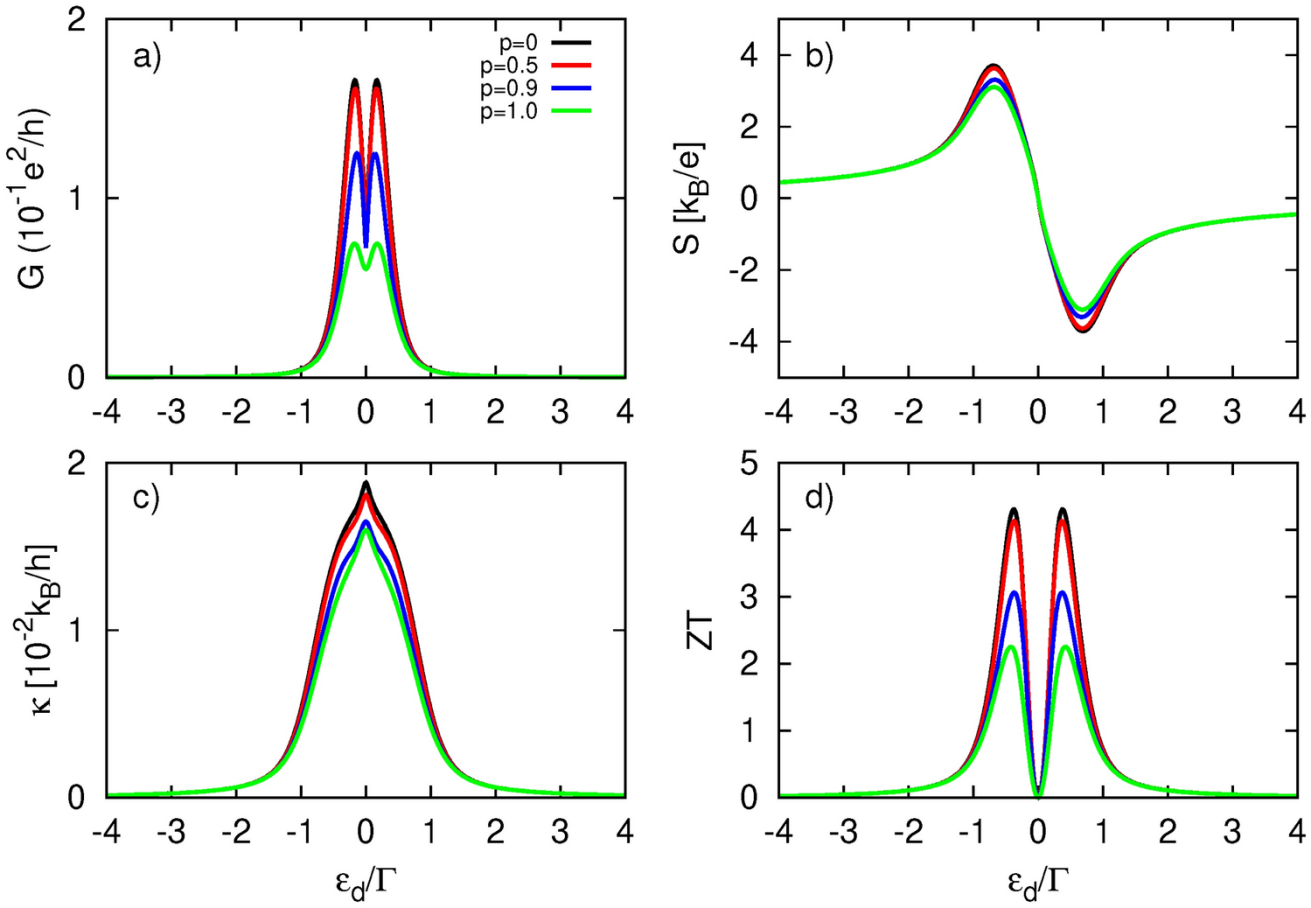
Thermoelectric coefficients: (**a**) electrical conductance, (**b**) Seebeck coefficient (thermopower), (**c**) heat conductance, (**d**) figure of merit, calculated as a function of the dot’s energy level $\varepsilon _d$ and for indicated values of spin polarization parameter *p*. The other parameters are: $\gamma _L=\gamma _R=0.1$, $k_B T=0.1\Gamma$, $U=0$.

In the following, we consider the situation where the external normal metal electrode is ferromagnetic, characterized by the corresponding spin-polarization factor *p*. To study the effect of the magnetism of the FM electrode on the thermoelectric response of the hybrid system, we present in Fig. 7 the thermoelectric coefficients calculated for various values of the spin-polarization factor. Electrical conductance, shown in Fig. 7a, directly follows the dependence of the transmission function on *p*, i. e. it decreases with increasing *p*. For $p=1$, *G* is half the value of *G* for an unpolarized left lead ($p=0$). When the left electrode is fully spin-polarized ($p=1$), only electrons with spin-up orientation contribute to the transport. Although the density of states of the FM lead for electrons with spin-up orientation increases with *p*, the number of states in the TI electrode remains the same for both spin components. Simultaneously, the spin-down component of the dot’s coupling to the ferromagnet, $\Gamma _L^\downarrow$, and the corresponding component of the density of states decrease with increasing *p*. As a result, *G* drops with increasing *p* and becomes halved for $p=1$ because only half of the TI’s electron states take part in the transport. The behavior of heat conductance, shown in Fig. 7c, can be explained in a similar manner. However, the rate of decrease for $\kappa$ is much smaller than that for *G*. In contrast, thermopower exhibits only a weak dependence on changes in the spin-polarization factor, acquiring roughly the same values for nearly every $\varepsilon _d$. Only close to the maxima of |*S*| does the thermopower reveal a slight drop with increasing *p*. This behavior can again be understood in terms of the competition between $L_0$ and $L_1$, as was the case for the coupling dependence. Finally, the figure of merit (Fig. 7d) decreases with increasing *p*, primarily due to the drop in electrical conductance. However, even with a fully spin-polarized left electrode, *ZT* still achieves remarkable values, exceeding 2. Comparing these results with the thermoelectric response dependence on the spin-polarization factor for QD system with normal metallic electrodes, as presented in Fig. 9 in the SI, one can again observe the advantages of the considered setup.

**Fig. 8:**
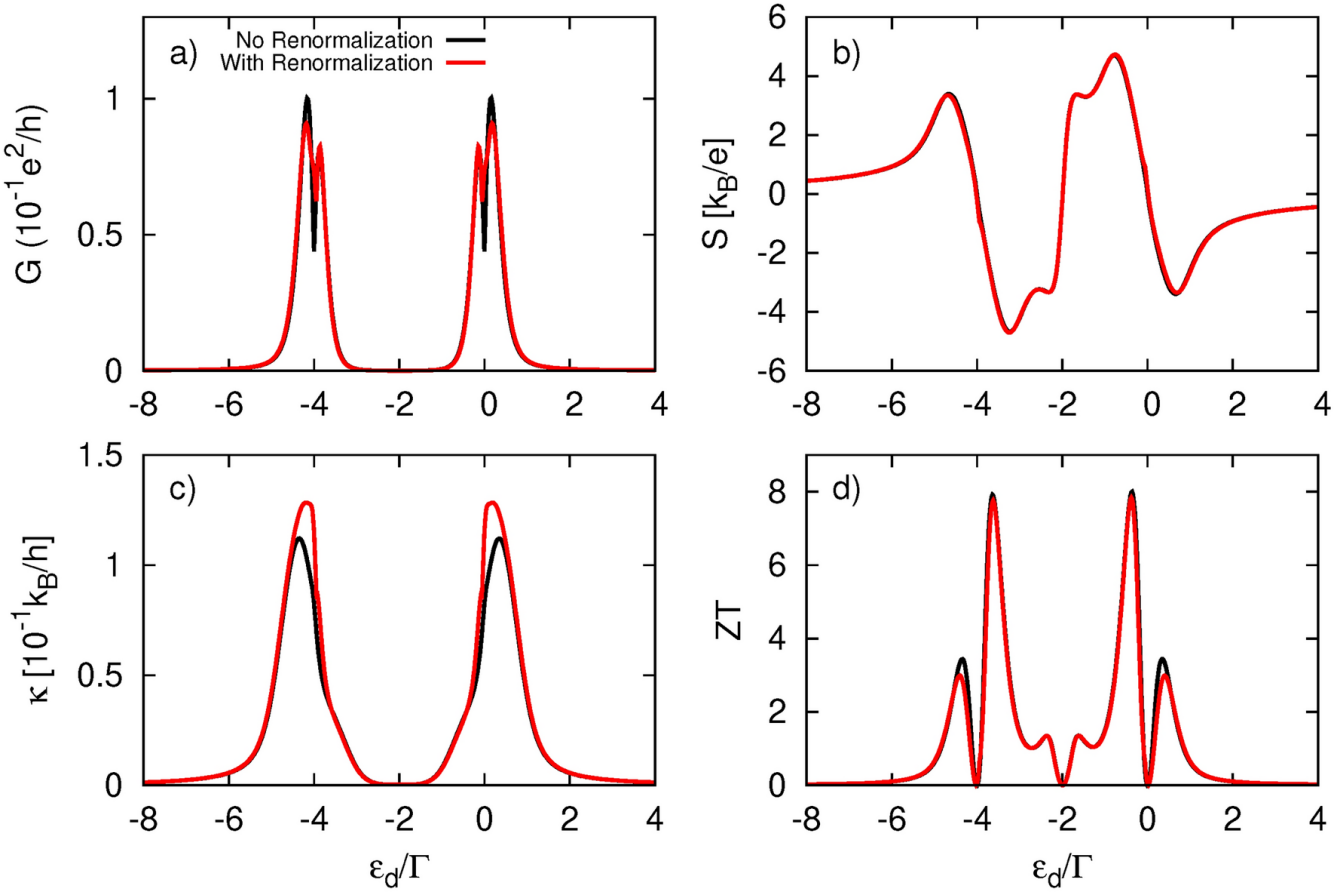
Thermoelectric coefficients: (**a**) electrical conductance, (**b**) Seebeck coefficient (thermopower), (**c**) heat conductance, (**d**) figure of merit, calculated as a function of the dot’s energy level $\varepsilon _d$ with and without inclusion of dot’s level renormalization. The other parameters are: $\gamma _L=\gamma _R=0.1$, $k_B T=0.1\Gamma$, $U=4\Gamma$, $p=0.9$.

To complete the study on conventional thermoelectricity, let us now consider the case of nonzero Coulomb repulsion in the dot, i. e. when *U* is finite. The corresponding numerical results are presented in Fig. 8 for two scenarios: with and without the inclusion of dot’s energy level renormalization due to spin-polarized tunneling processes occurring between the QD and the FM electrode. The renormalization effects can be accounted for by considering an effective exchange field $\Delta B$, which affects the dot’s energy level in a manner similar to that of an external magnetic field, lifting its degeneracy and leading to the spin-split dot’s energy level:

32 $$\begin{aligned} \varepsilon _{d\sigma }=\varepsilon _d+\tilde{\sigma }\frac{\Delta B}{2} \end{aligned}$$

with

33 $$\begin{aligned} \Delta B=(\Gamma _L^{\uparrow }-\Gamma _L^{\downarrow })[F(\varepsilon _d)-F(\varepsilon _d+U)] \end{aligned}$$

calculated within second order perturbation theory. Here, $F(x)=(1/(2\pi )) \Re [\psi [1/2 + i (x - \mu _L)/(2\pi k_BT)]]$ and $\psi (x)$ denotes polygamma function. Note that exchange field is a linear function of *p*.

First, we briefly describe the differences in thermoelectric response caused by Coulomb interactions. For an exact comparison, the results presented here were obtained using the same remaining parameters as those displayed in Fig.8 for $p=0.9$. The electrical conductance (Fig.8a) exhibits a doubled two-peak structure. One of the resonances is located at $\varepsilon _d=0$, similar to the $U=0$ case,while its Coulomb counterpart is situated at $\varepsilon _d=-U$. It is important to note that the dot’s coupling to the TI is not strong enough to induce the renormalization effect due to the real part of the self-energy $\Sigma _{R\sigma }^r$; thus, the positions of the resonances strictly follow the poles of the bare dot’s Green function. Furthermore, the two-peak structures are not symmetric as they are in the $U=0$ case, and the amplitudes are slightly attenuated. In turn, the Seebeck coefficient vanishes now at three points: two correspond to resonances at $\varepsilon _d=-U,0$, while the third is the particle-hole symmetry point at $\varepsilon _d=-U/2$. Moreover, finite Coulomb interactions leads to an enhancement of |*S*|. The heat conductance $\kappa$, shown in Fig. 8c, follows the electrical conductance, revealing two peaks located nearby at $\varepsilon _d=-U,0$, with their amplitudes also reduced compared to the $U=0$ situation. However, heat conductance does not exhibit the central peak situated at $\varepsilon _d=-U/2$, which is usually attributed to the bipolar effect^45,64^. The absence of this feature can be associated with an insufficiently high temperature. Since the two resonances are far apart, more energy is needed to drive the bipolar effect. Indeed, at higher temperatures, the central peak in $\kappa$ appears, as shown in Fig. 4 in the Supplementary Information. The most significant impact of Coulomb repulsion can be noticed in the figure of merit displayed in Fig. 8d. In addition to the richer peak structure, there is a remarkable doubling o *ZT* values for certain dot energy levels, which can now reach up to 8.

**Fig. 9:**
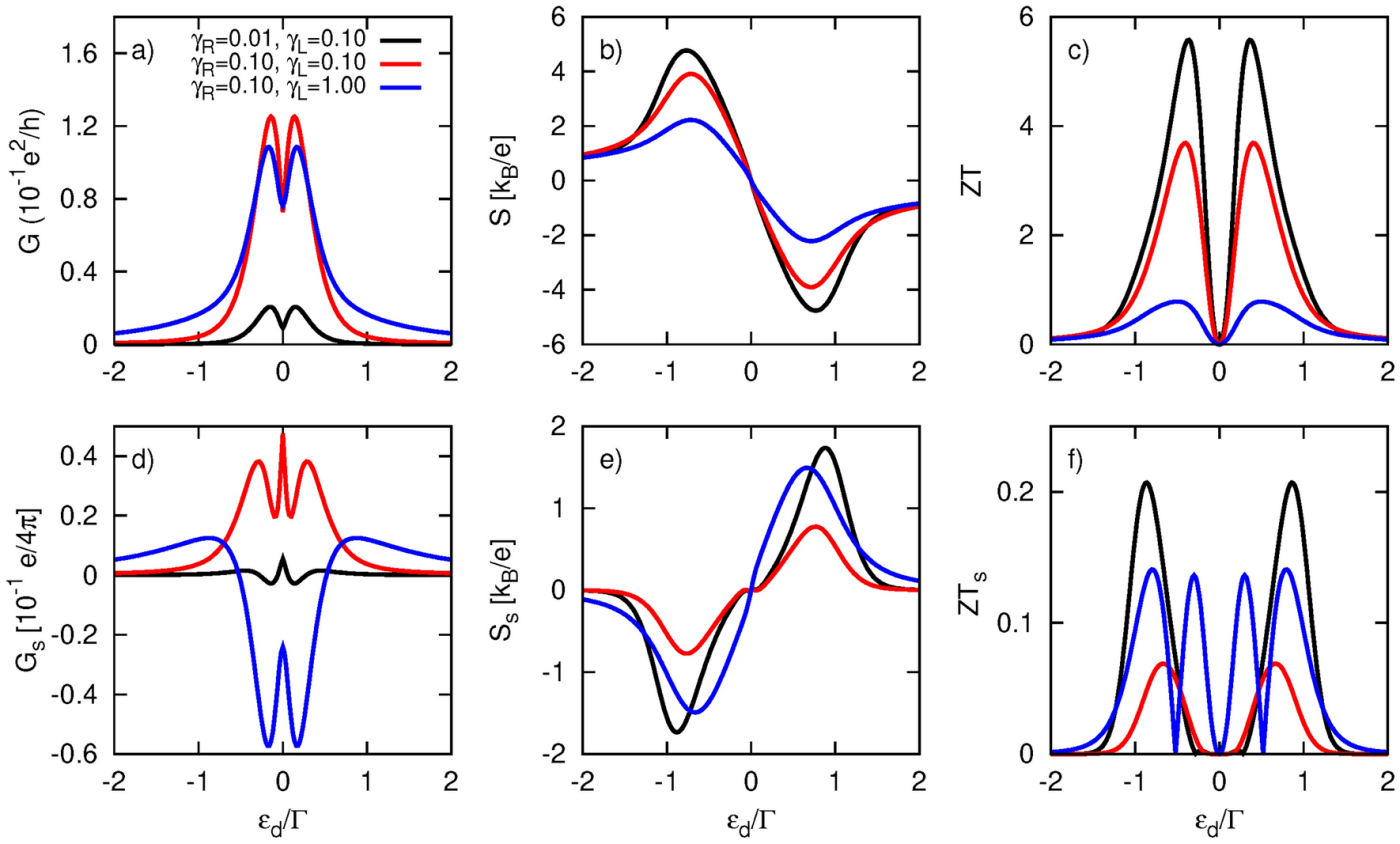
Thermoelectric coefficients: (**a**) electrical conductance, (**b**) Seebeck coefficient (thermopower), (**c**) figure of merit, (**d**) spin conductance, (**e**) spin thermopower, (**f**) spin figure of merit, calculated as a function of the dot’s energy level $\varepsilon _d$ and for indicated values of coupling parameters $\gamma _L$ and $\gamma _R$, and for $p=0.9$. The other parameters are as in Fig. 4.

Now, we briefly analyze the impact of dot level renormalization due to the ferromagnetism of the left electrode. It appears that the splitting of the dot’s level by the exchange field has only a marginal influence on the thermoelectric coefficients in this coupling regime, even though we assumed a relatively large spin polarization factor. Only the electrical conductance *G* (which becomes slightly suppressed) and the heat conductance $\kappa$ (which becomes slightly enhanced) are affected, while the Seebeck coefficient *S* remains unchanged. As a result, the figure of merit *ZT* is almost unaffected by the exchange field.

### Spin thermoelectric effects

Here, we consider the situation in which spin accumulation in the ferromagnetic electrode is present, allowing for a spin thermoelectric response. In Fig. 9, we show both charge thermoelectric coefficients (*G*, *S*, *ZT*) and spin thermoelectric coefficients ($G_s$, $S_s$, $ZT_s$), all calculated under the condition of vanishing spin current, which defines spin thermoelectricity. Since the behavior of the charge thermoelectric coefficients is similar to that observed in the absence of spin bias, we will focus here on the spin thermoelectric quantities.

In particular, the spin conductance $G_s$ is positive regardless of the dot’s energy level for $\gamma _R\ge \gamma _L$. However, it can also acquire negative values if only $\gamma _R<\gamma _L$. Thus, by tuning the dot’s couplings, one can switch the sign of $G_s$ for a given $\varepsilon _d$, indicating the possibility of changing the direction of the spin current. This feature is significant for potential applications. Spin thermopower (Fig. 9e) resembles thermopower (Fig. 9b) but taken with the opposite sign. The opposite sign of $S_s$ compared to *S* arises from the relationship between the spin-resolved thermopowers, which have the same sign for a given $\varepsilon _d$, while also satisfying $|S_{\uparrow }|<|S_{\downarrow }|$ for $p>0$. The latter feature can be explained by considering the enhancement (suppression) of the spin resolved conductance $G_\uparrow$ ($G_\downarrow$) with increasing *p* (see also Fig. 6d,e in the SI), in conjunction with the condition under which $S_\sigma$ is calculated i. e. the vanishing charge current in the spin $\sigma$ channel. As a result, a larger bias voltage must be applied to compensate for the thermally generated current in the spin $\downarrow$ channel. The corresponding spin figure of merit is shown in Fig. 9f. Both *ZT* and $ZT_s$ can be optimized by tuning the tunnel couplings. Moreover, the spin thermoelectric response can be enhanced by increasing the spin-polarization factor *p* of the FM electrode, as shown in Fig. 6 in the SI. Specifically, spin thermoelectric coefficients, $G_s$, $|S_s|$, and $ZT_s$, monotonically increase with *p*, achieving their maximum values as $p \rightarrow 1$. Conversely, conventional thermoelectric quantities, *G* and *ZT*, exhibit a dependence similar to that presented in Fig.7, except for |*S*|, which slightly increases with *p*, whereas |*S*| in Fig.7 slightly decreases with *p*. This discrepancy arises from the different conditions under which these quantities were calculated. Recall that the coefficients shown in Fig.7 were obtained under the condition of vanishing charge current, while those presented in Fig. 6 in the SI and Fig. 9 were derived under the condition of vanishing both spin and charge currents.

## Conclusions

In conclusion, we have investigated the thermoelectric response of a quantum dot coupled to a ferromagnetic electrode and attached to a topological insulator. By employing the Green’s function technique, we determined the electrical and heat currents flowing through the system and derived the relevant linear response thermoelectric coefficients, both in the absence and presence of spin accumulation in the ferromagnetic lead.

Our analysis began with a study of the transmission coefficients to gain a deeper understanding of the forthcoming thermoelectric response of the system. We demonstrated that a sufficiently strong coupling between the quantum dot and the surface states of the topological insulator leads to the renormalization of the dot’s energy level. Additionally, we found that the width of the transmission feature is influenced by the dot’s coupling to both the ferromagnetic and topological insulator electrodes.

Based on the transmission results, we provided a detailed analysis of the transport and thermoelectric coefficients, including electrical and heat conductance, thermopower, figure of merit, and their relevant spin counterparts. In particular, both electrical and heat conductances exhibit a nonmonotonic dependence on coupling to the ferromagnetic and topological insulator electrodes. The Seebeck coefficient demonstrates a more complex relationship with the dot’s coupling to the topological insulator, while it decreases with increasing coupling strength to the ferromagnetic lead.

Additionally, we have shown that the figure of merit, which indicates the thermoelectric efficiency of the system, can achieve large values by tuning the coupling strengths. The spin polarization of the ferromagnetic electrode further impacts the thermoelectric response. Coulomb interactions on the dot lead to an additional enhancement of thermoelectric efficiency, whereas the renormalization of the dot’s level due to spin-dependent coupling with the ferromagnetic electrode has only a minor effect on the thermoelectric response in the considered coupling regime.

Finally, we investigated spin thermoelectric effects and demonstrated that the spin conductance can switch sign by adjusting the coupling strengths, paving the way for potential applications in magnetic memory technologies.

## Supplementary Information

Supplementary Information.

## Data Availability

The datasets used and/or analysed during the current study available from the corresponding author on reasonable request.
