# Supplementary material for: Spin-dependent thermoelectric properties of a hybrid ferromagnetic metal/quantum dot/topological insulator junction
Source: Sci Rep. 2025 Feb 10;15:4904. doi: 10.1038/s41598-025-87931-7 (PMC11811068; doi:10.1038/s41598-025-87931-7)
Supplement: Supplementary file 1 — Supplementary Information. [file 41598_2025_87931_MOESM1_ESM.pdf]

Spin-dependent thermoelectric properties of a hybrid ferromagnetic metal/quantum dot/topological insulator junction

Piotr Trocha[1,*]

[1]Institute of Spintronics and Quantum Information, Faculty of Physics and Astronomy, Adam Mickiewicz University, Poznań, 61-614, Poland
[*]ptrocha@amu.edu.pl

Additional figures for transmission and thermoelectric coefficients

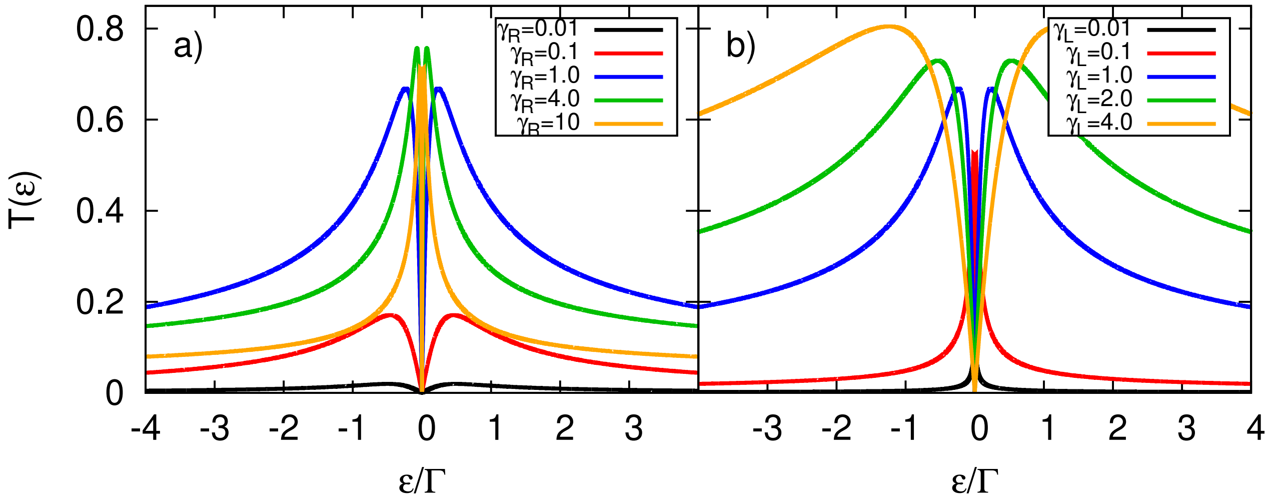

Figure 1. Transmission coefficients as a function of energy calculated for indicated values of the coupling parameters and for $\varepsilon_d = 0$. Left panel a) shows results for $\gamma_L = 1$ while varying γ_R, whereas right panel b) presents situation for $\gamma_R = 1$ while varying $= \gamma_L$. Other parameters: $U = 0$, $p = 0$, $T = 0$.

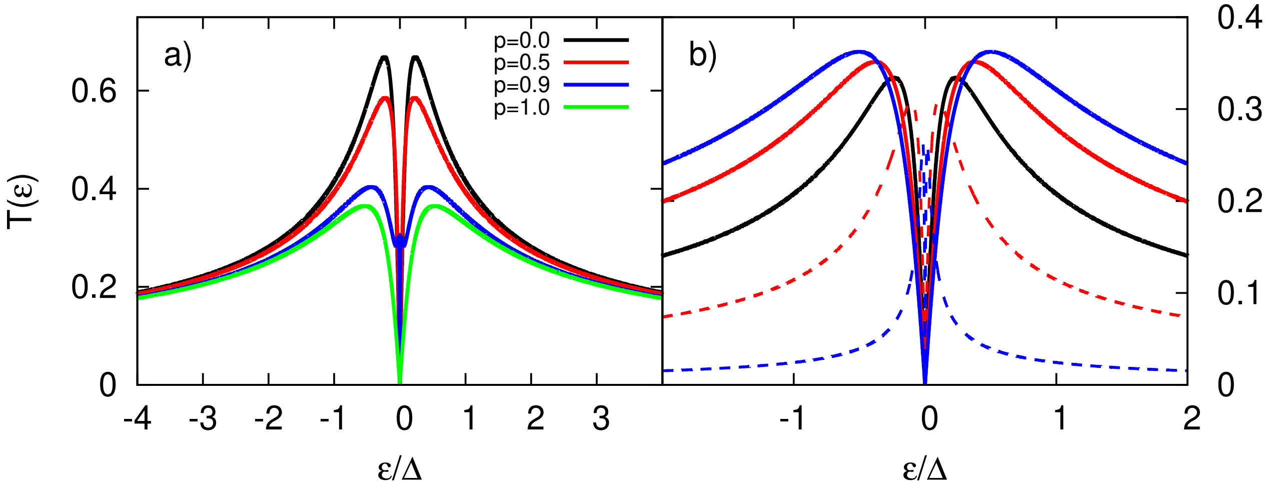

Figure 2. Transmission coefficients as a function of energy calculated for indicated values of spin polarization factor p and for $\varepsilon_d = 0$. Left panel a) shows total transmission, whereas right panel b) displays spin up (solid line) and spin down (dashed line) transmission coefficients. Other parameters: $\gamma_R = 1$, $U = 0$, $T = 0$.

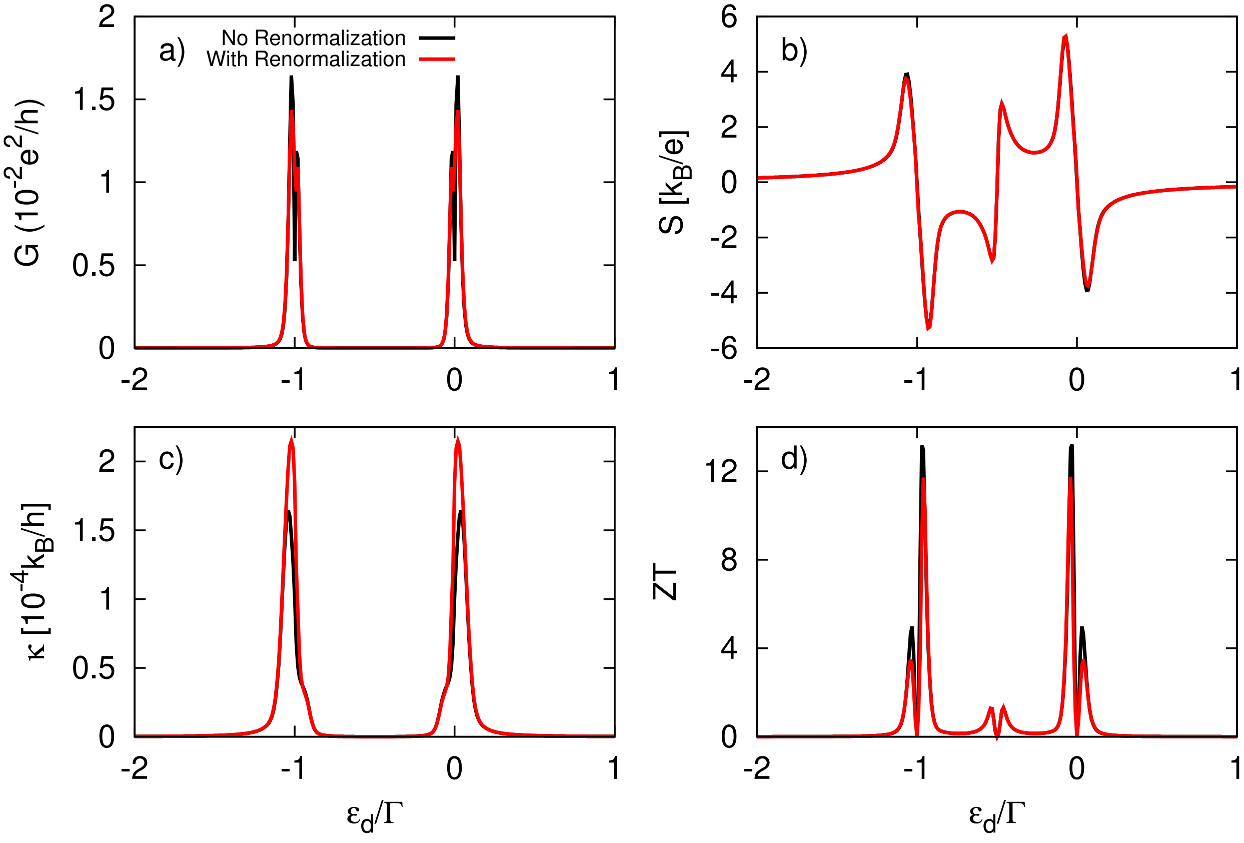

Figure 3. Thermoelectric coefficients: a) electrical conductance, b) Seebeck coefficient (thermopower), c) heat conductance, d) figure of merit, calculated as a function of the dot's energy level ε_d with and without inclusion of dot's level renormalization. The other parameters are: $\gamma_L = \gamma_R = 0.01$, $k_B T = 0.01\Gamma$, $U = \Gamma$, $p = 0.9$.

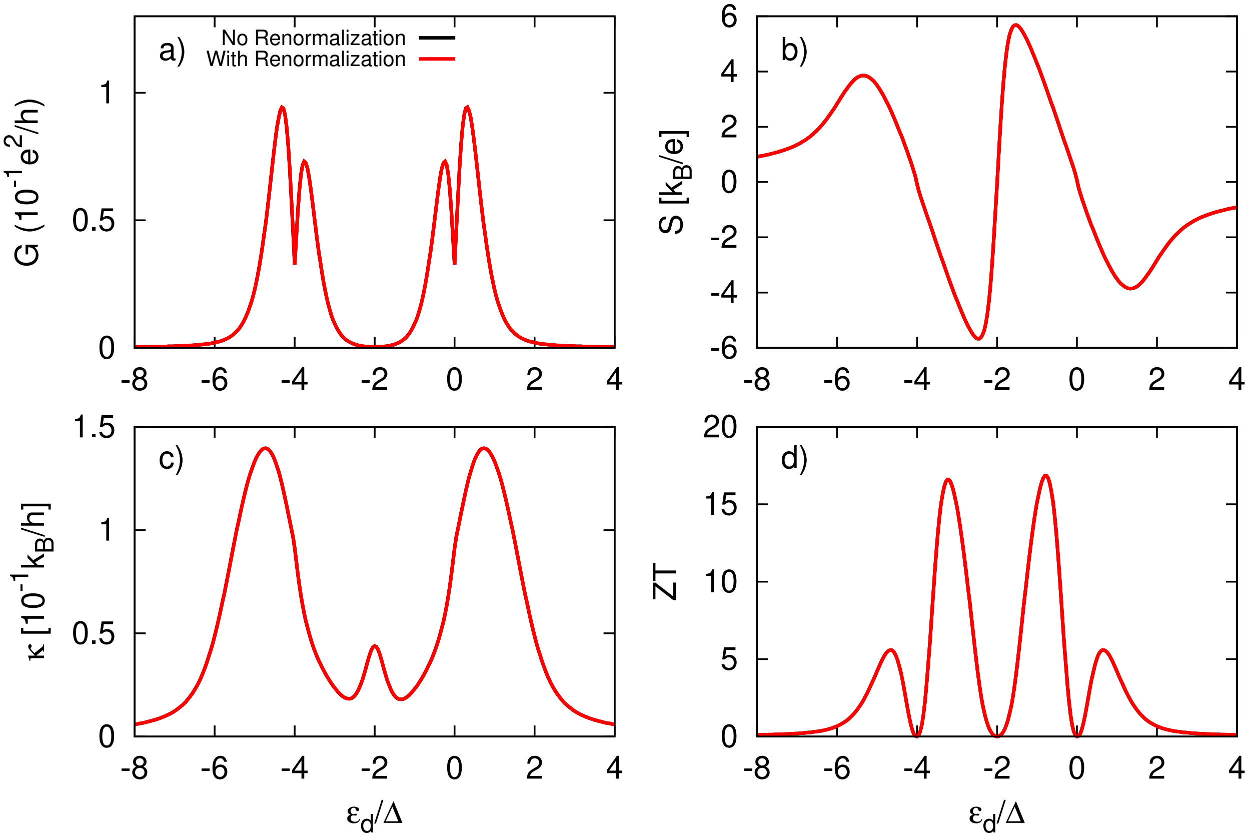

Figure 4. Thermoelectric coefficients: a) electrical conductance, b) Seebeck coefficient (thermopower), c) heat conductance, d) figure of merit, calculated as a function of the dot's energy level ε_d with and without inclusion of dot's level renormalization. The other parameters are: $\gamma_L = \gamma_R = 0.1$, $k_B T = 0.2\Gamma$, $U = 4\Gamma$, $p = 0.9$.

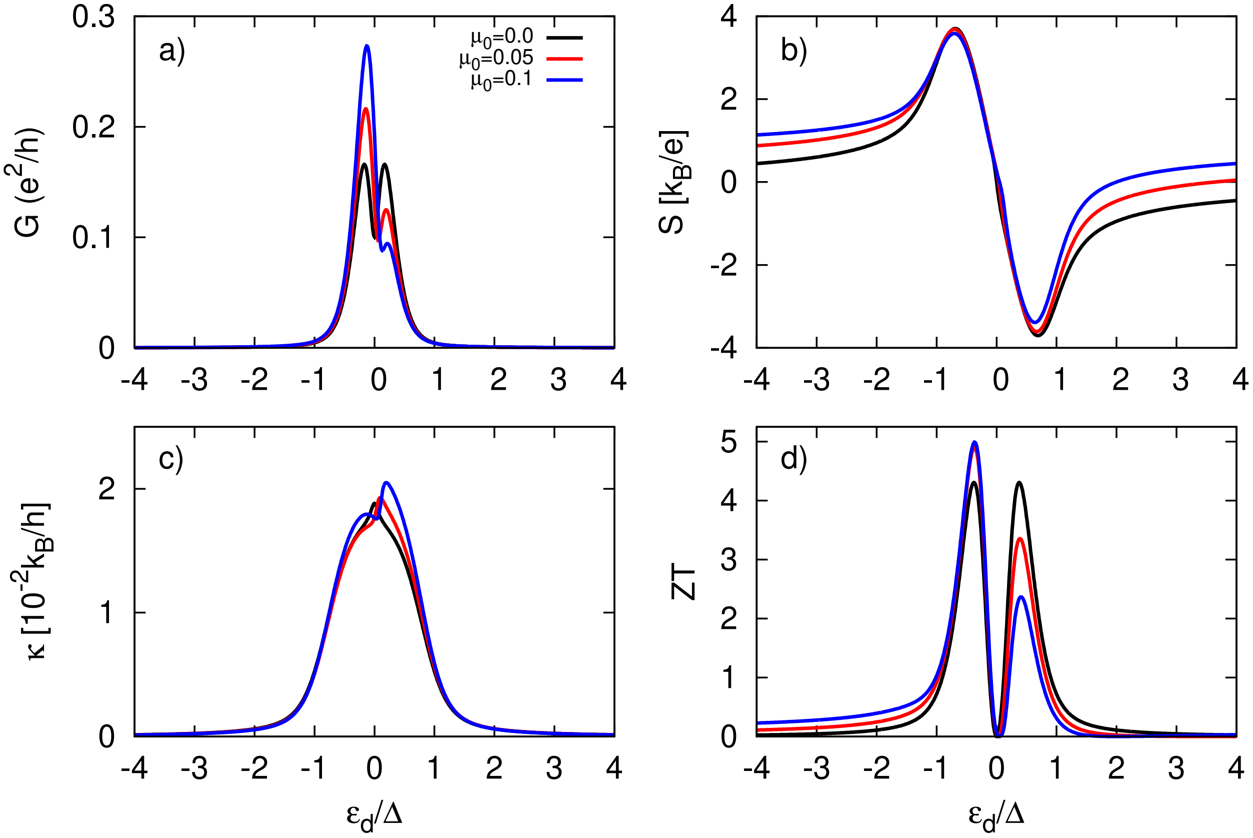

Figure 5. Thermoelectric coefficients: a) electrical conductance, b) Seebeck coefficient (thermopower), c) heat conductance, d) figure of merit, calculated as a function of the dot's energy level ε_d calculated for indicated values of the position of the Dirac point from Fermi level. The other parameters are: $\gamma_L = \gamma_R = 0.1$, $k_BT = 0.1\Gamma$, $U = 0$, $p = 0$.

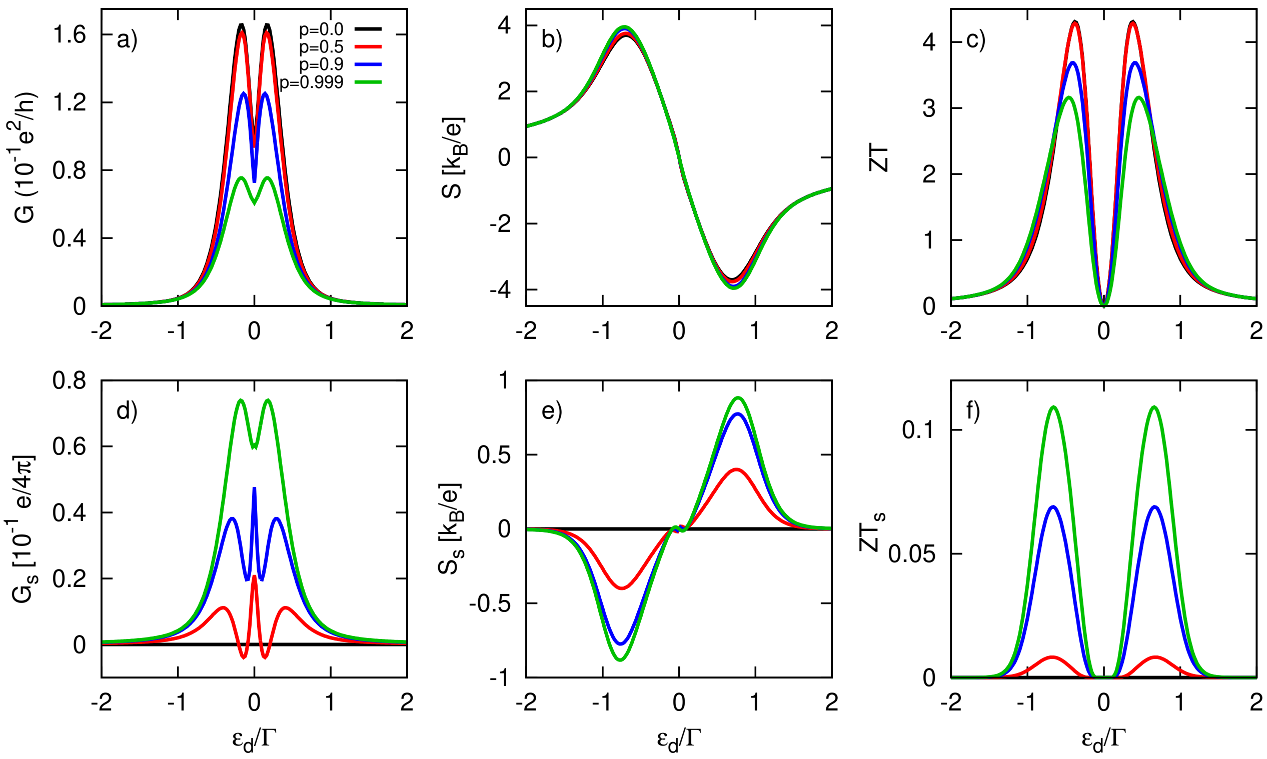

Figure 6. Thermoelectric coefficients: a) electrical conductance, b) Seebeck coefficient (thermopower), c) figure of merit, d) spin conductance, e) spin thermopower, f) spin figure of merit, calculated as a function of the dot's energy level ε_d and for indicated values of spin-polarization factor p. The other parameters are: $\gamma_L = \gamma_R = 0.1$, $k_B T = 0.1\Gamma$, $U = 0$.

Thermoelectric coefficients for QD coupled to normal metal leads

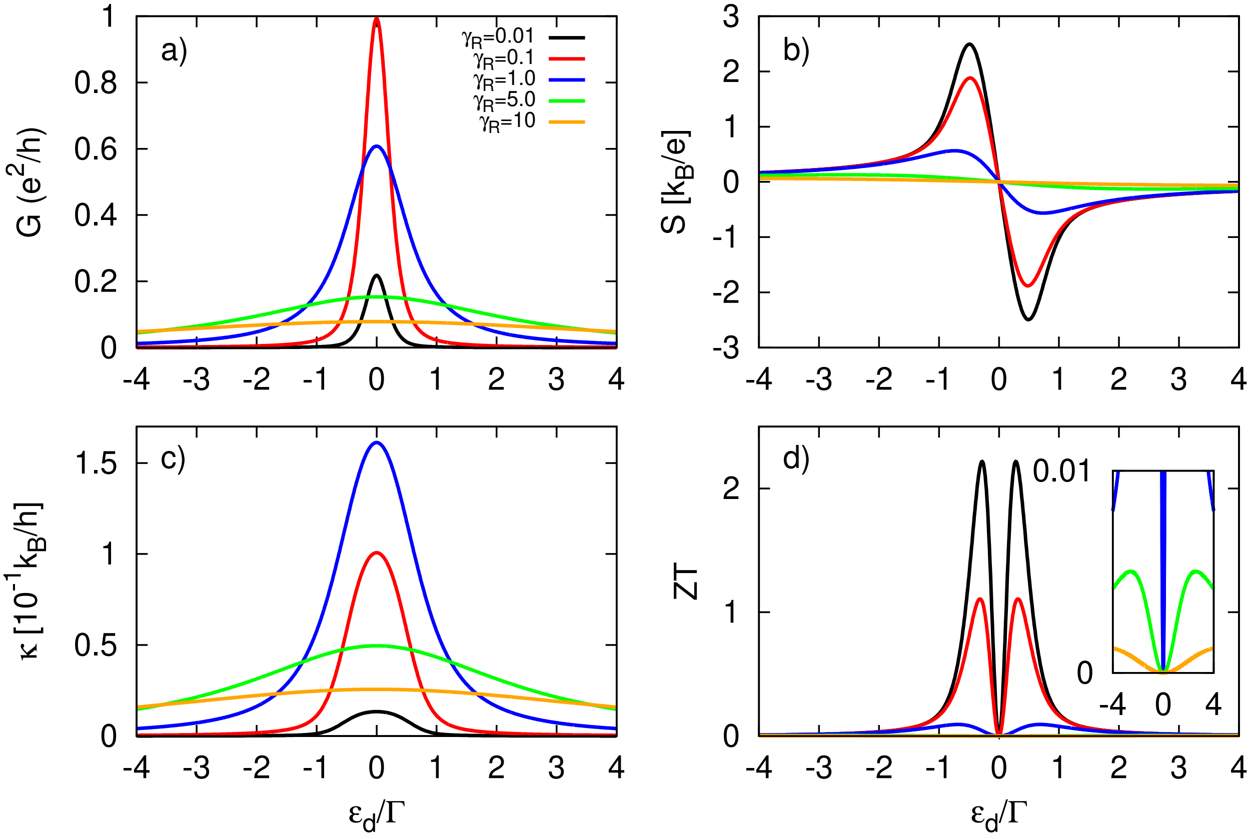

Figure 7. Thermoelectric coefficients for NM-QD-NM system: a) electrical conductance, b) Seebeck coefficient (thermopower), c) heat conductance, d) figure of merit, calculated as a function of the dot's energy level ε_d calculated for indicated values of the parameter γ_R. Inset in d) shows a zoomed-in view of the central peaks region; for clarity only curves for $\gamma_R = 1, 5, 10$ are displayed. The other parameters are: $\gamma_L = 0.1$, $k_B T = 0.1\Gamma$, $U = 0$, $p = 0$.

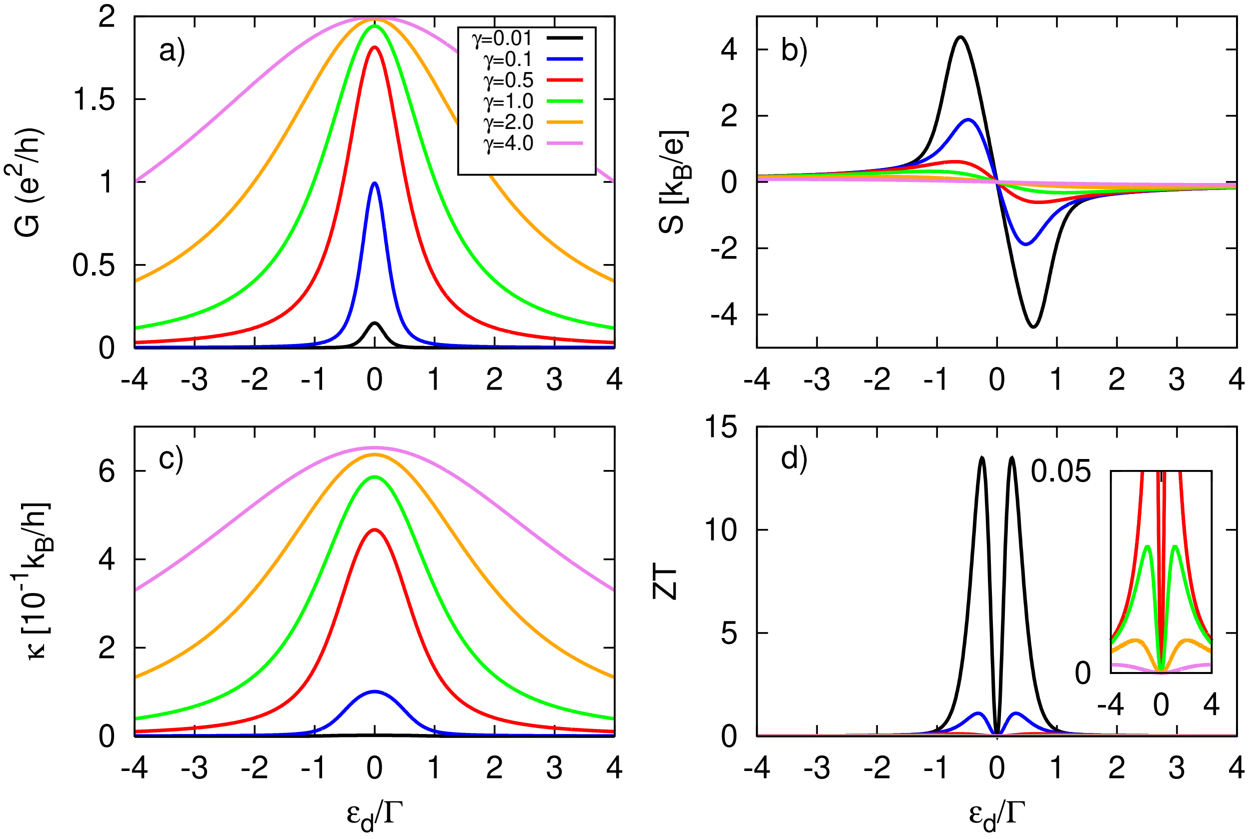

Figure 8. Thermoelectric coefficients for NM-QD-NM system: a) electrical conductance, b) Seebeck coefficient (thermopower), c) heat conductance, d) figure of merit, calculated as a function of the dot's energy level ε_d calculated for indicated values of the parameter $\gamma = \gamma_L = \gamma_R$. Inset in d) shows a zoomed-in view of the central peaks region; for clarity only curves for $\gamma = 0.5, 1, 2, 4$ are displayed. The other parameters are: $k_B T = 0.1\Gamma$, $U = 0$, $p = 0$.

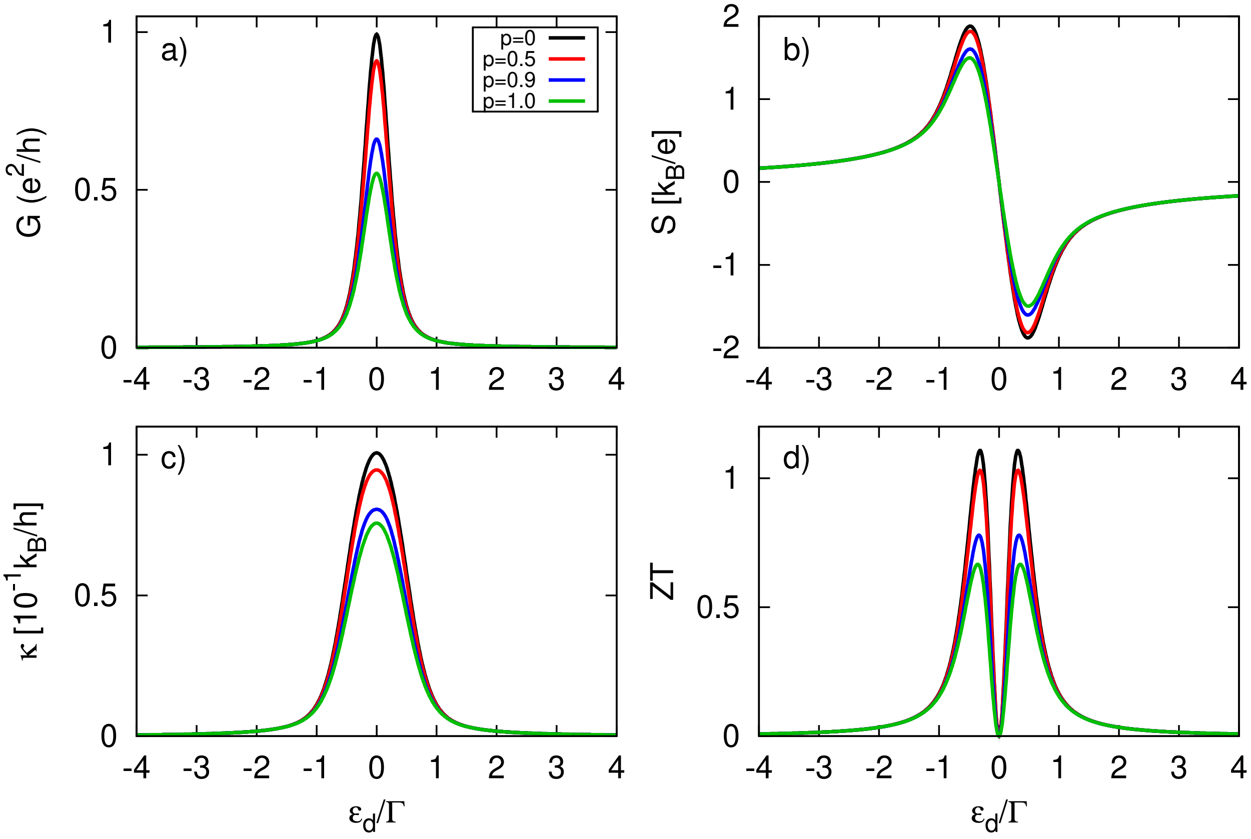

Figure 9. Thermoelectric coefficients for FM-QD-NM system with FM being metallic ferromagnet when $p > 0$: a) electrical conductance, b) Seebeck coefficient (thermopower), c) heat conductance, d) figure of merit, calculated as a function of the dot's energy level ε_d calculated for indicated values of the spin-polarization factor p. The other parameters are: $k_B T = 0.1\Gamma$, $U = 0$, $p = 0$.
